# The telomere bouquet facilitates meiotic prophase progression and exit in fission yeast

**DOI:** 10.1038/celldisc.2017.41

**Published:** 2017-11-07

**Authors:** Vera Moiseeva, Hanna Amelina, Laura C Collopy, Christine A Armstrong, Siân R Pearson, Kazunori Tomita

**Affiliations:** 1 Chromosome Maintenance Group, UCL Cancer Institute, University College London, London, UK

**Keywords:** telomeres, meiosis, *S. pombe*, chromosome, bouquet, Cdc2-Cdc13, Rad3–Chk1, the LINC complex

## Abstract

During meiotic prophase, chromosome arrangement and oscillation promote the pairing of homologous chromosomes for meiotic recombination. This dramatic movement involves clustering of telomeres at the nuclear membrane to form the so-called telomere bouquet. In fission yeast, the telomere bouquet is formed near the spindle pole body (SPB), which is the microtubule organising centre, functionally equivalent to the metazoan centrosome. Disruption of bouquet configuration impedes homologous chromosome pairing, meiotic recombination and spindle formation. Here, we demonstrate that the bouquet is maintained throughout meiotic prophase and promotes timely prophase exit in fission yeast. Persistent DNA damages, induced during meiotic recombination, activate the Rad3 and Chk1 DNA damage checkpoint kinases and extend the bouquet stage beyond the chromosome oscillation period. The auxin-inducible degron system demonstrated that premature termination of the bouquet stage leads to severe extension of prophase and consequently spindle formation defects. However, this delayed exit from meiotic prophase was not caused by residual DNA damage. Rather, loss of chromosome contact with the SPB caused delayed accumulation of CDK1-cyclin B at the SPB, which correlated with impaired SPB separation. In the absence of the bouquet, CDK1-cyclin B localised near the telomeres but not at the SPB at the later stage of meiotic prophase. Thus, bouquet configuration is maintained throughout meiotic prophase, by which this spatial organisation may facilitate local and timely activation of CDK1 near the SPB. Our findings illustrate that chromosome contact with the nuclear membrane synchronises meiotic progression of the nucleoplasmic chromosomes with that of the cytoplasmic SPB.

## Introduction

Meiosis is an essential process for the generation of genetic diversity. In early meiotic prophase, telomeres cluster at the nuclear membrane. The term chromosomal or telomere bouquet was coined because this polarised chromosome arrangement resembles a bunch of flowers. The bouquet was first described a century ago and similar arrangements have been observed in diverse eukaryotic organisms [1, 2]. Despite evolutionary conservation of the bouquet configuration, its function appears to be diverse and is slowly being elucidated in each organism [3].

The bouquet configuration is achieved by interaction of chromosomes with the linker of nucleoskeleton and cytoskeleton (LINC) complex that interacts with motor proteins, promoting dynamic movement of chromosomes. As expected from its structure, bouquet formation restricts chromosome mobility at telomeres and telomere-led chromosome oscillation promotes pairing of homologous regions followed by synapsis and meiotic recombination between homologues [4, 5]. In some organisms chromosomes form contacts with the LINC complex *via* the centromeres or specific domains called pairing centres [5–7]. In mammals, this anchoring of telomeres as a bouquet, and the dynamic movement of chromosomes, are both essential processes for gametogenesis, suggesting a crucial role for the bouquet in meiotic progression [8–16].

Association of chromosomes with the LINC complex is controlled by meiotic progression. At the chromosome oscillation stage, meiotic recombination and synaptonemal complex formation between homologous chromosomes are initiated. The duration of the bouquet stage varies among organisms but termination of the bouquet configuration seems to be associated with chromosomal events. Association of chromosomes with the LINC complex is resolved before the pachytene stage, where homologues are stabilised by the completion of synapsis and/or establishment of chiasmata [5, 17, 18]. The ‘pachytene checkpoint’ monitors synapsis and meiotic recombination to ensure preparation of chromosomes for two sequential chromosome segregations [19, 20].

In fission yeast, the telomere bouquet is observed as a tightly focused cluster of telomeres on the nuclear membrane close to the SPB [21]. In this species, bouquet formation begins when the meiosis-specific telomere proteins Bqt1 and Bqt2 are expressed before pre-meiotic S-phase [22–24]. The Bqt1–Bqt2 heterodimer interacts with the telomeric DNA-binding protein complex Taz1-Rap1. Bqt1 on its own interacts with the inner nuclear membrane protein Sad1 (a component of the LINC complex). The meiotic telomere-associated LINC complex migrates toward the cytoplasmic SPB to form the telomere bouquet [22, 25]. Once the bouquet is formed, the chromosomes oscillate *via* drastic movement of the SPB. The nucleus is pulled into an elongated shape and oscillates back and forth, led by the telomeres and the SPB; this oscillation period is defined as the ‘horsetail stage’ [21, 26, 27]. Bouquet formation is terminated when telomeres disperse and dissociate from the SPB in a phenomenon termed ‘telomere fireworks’ [28].

Elimination of any bouquet component disrupts the formation of the bouquet and impairs the efficiency of homologous chromosome alignment and mei tic recombination in fission yeast [22–26, 29, 30]. However, the bouquet arrangement plays a further role in enabling the SPB to form a functional spindle [28, 31]. Spindle formation requires insertion of the SPB into the nucleoplasmic side of the nuclear membrane *via* local breakdown of the membrane, a process called *fenestration* [32]. In cells harbouring mutations within the bouquet proteins, the nuclear membrane around the SPB fails to resolve, thereby blocking the formation of meiotic spindles [33]. Such defective SPBs fail to accumulate Sad1 and detach from the nuclear membrane [28, 34]. Meiotic centromere formation is also impaired in the absence of bouquet formation [35]. Bouquet formation releases centromeres from the SPB, following which the kinetochores and the SPB are partly dismantled during meiotic prophase. However, prior to meiosis I, the meiotic kinetochores and the SPB are reconstructed or ‘matured’ [36–39]. Thus, *via* bouquet formation, the meiotic telomere influences meiotic processes at other chromosomal regions and at the SPB. Nevertheless, how chromosomes communicate with the nuclear membrane and the SPB remains to be established.

Before meiotic chromosome segregation, the chromosomes are replicated and homologues recombine to establish chiasmata. This chromosome ‘maturation’ process is essential for faithful chromosome segregation during meiosis. Meiotic recombination is initiated *via* expression of Rec12, the meiosis-specific nuclease (the SPO11 homologue in fission yeast), and meiosis-specific homologous recombination pathways engage cross-over recombination between homologous chromosomes [3]. In fission yeast, these processes and entry into meiosis are controlled by CDK1 activity and expression of Mei4, a meiosis-specific transcription factor [40–44]. Impaired meiotic replication caused by a stalled replication fork or lack of dNTPs is mainly detected by the DNA damage checkpoint protein, Cds1, which represses CDK1 activity [42, 45]. Cds1 also suppresses transcription of Mei4, which is required for expression of Rec12 and the Cdc25 phosphatase that counteracts the activity of Wee1, the CDK1 repressor kinase. Thus, when meiotic replication is impaired, cells arrest before the meiotic recombination stage [43, 44]. Expression of Mei4 also leads to deceleration of chromosome movement, thereby terminating the horsetail stage [45, 46]. Impaired DNA repair during meiotic recombination is mainly detected by the meiosis-specific checkpoint kinase Mek1, which represses Cdc25 [47]. Both Cds1 and Mek1 are expressed in early meiotic prophase and are activated by Rad3 (the ATR homologue) and other ‘Rad’ DNA damage checkpoint proteins [45, 48]. Rad3 also controls another kinase, Chk1, which activates Wee1 [45]. However, expression of Chk1 appears to occur later during meiotic prophase [48], and its functional significance in meiosis is not yet known.

Fission yeasts do not engage in synaptonemal complex formation, and therefore there is no obvious ‘pachytene checkpoint’ during meiotic prophase. Hence, cells enter the chromosome segregation phase immediately after the meiotic recombination stage. Here, we have investigated the timing of bouquet termination and the progression of meiosis in fission yeast. Live single-cell imaging elucidated that remaining DNA damages from meiotic recombination activate Rad3 and Chk1 to restrain bouquet formation and meiotic prophase. Crucially, CDK1-cyclin B localises near the telomeres at later stages of meiotic prophase so that meiotic telomeres facilitate SPB activation *via* bouquet configuration. Thus, we conclude that the telomere–LINC complex connects the timing of chromosome maturation in the nucleus with the timing of SPB maturation in the cytoplasm to promote exit from prophase.

## Results

### The bouquet stage is retained under impaired meiotic recombination

**Figure 1:**
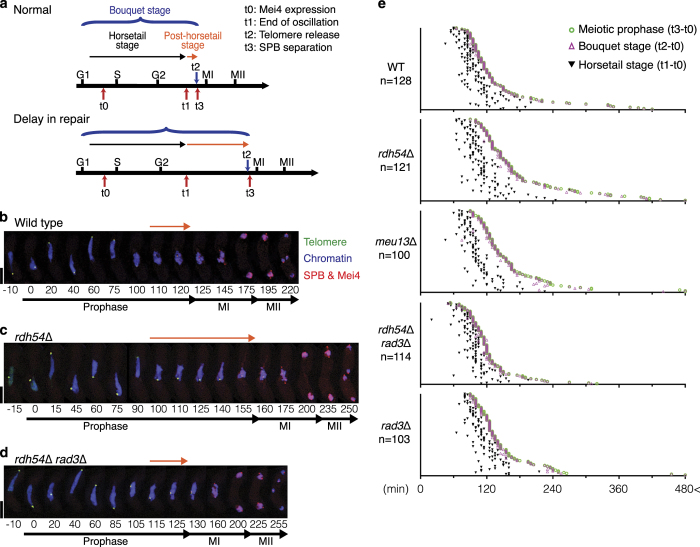
Live imaging of fission yeast meiosis and measurement of bouquet stage extension. (**a**) Schematic diagram of meiotic progression. t0, t1, t2 and t3 indicate time of the beginning of Mei4 expression, the end of chromosome oscillation, the beginning of telomere release (the end of bouquet configuration) and SPB separation, respectively. The chromosomal bouquet is observed throughout meiotic prophase (highlighted with a blue curved bracket). This stage can be divided into the ‘horsetail stage’ (black arrow line flanked by t0 and t1) and the ‘post-horsetail stage’ (orange arrow line flanked by t1 and t3). Delayed DNA repair extends the post-horsetail stage within the bouquet stage. (**b**–**d**) Series of frames from films of live fission yeast undergoing meiosis (**b**: wild type, **c**: *rdh54*Δ, D: *rdh54*Δ *rad3*Δ). The SPB, Mei4, telomeres and chromosomes were visualised *via* endogenously tagged Sid4-mCherry, Mei4-mCherry, Taz1-YFP and Hht1-Cerulean, respectively. Merged images are presented. Individual channels are shown in Supplementary Figure S2. Cell images were captured every 5 min, and selected time frames are shown. Numbers below the slides represent minutes since Mei4 stains nuclei (t0: see details in Supplementary Figure S1C). The end of meiotic prophase (t3) is defined by SPB separation and chromosome condensation. The period between t0 and t3 is defined as the 'meiotic prophase' in this system. The post-horsetail stage is highlighted with the orange arrow lines. Scale bars equal 5 μm. (**b**) An example of average wild-type meiosis. Telomeres cluster at the SPB, representing bouquet formation, during meiotic prophase SPB oscillation pulls chromosomes to exhibit chromosomal ‘horsetail’ movement until 110 min (t1). The SPB settles at the centre of a cell, and separates when telomeres dissociate from the SPB and disperse (telomere fireworks, t2: 125 min). (**c**) *rdh54*Δ cells extend the post-horsetail stage (t1: 100 min). Telomere dissociation is observed at 160 min (t2) when the SPB separates (t3). The SPB segregates equally twice through meiosis; however, chromosome segregation is defective. (**d**) *rdh54*Δ *rad3*Δ cells exhibit shortened post-horsetail stage and telomere dissociation (t2) is observed at 130 min. (**e**) The duration of the events for individual cells are shown. Dot plots represent time distributions of meiotic prophase [t3–t0] (green circle), the bouquet stage [t2–t0] (magenta triangle) and the horsetail stage [t1–t0] (black anti-triangle). Up to 20 cells were filmed per day and the total examined samples are summarised. The sample number (*n*=) is indicated below the genotype. For values in each sample, see Supplementary Table S2.

In order to determine the timing of bouquet termination using single-cell live imaging of homothallic *h*^*90*^ zygote meiosis, we devised an experimental system to allow us to visualise all major nuclear meiotic events. Telomeres and chromosomes were visualised with Taz1-YFP and Hht1 (histone H3)-Cerulean, respectively, and both the SPB and Mei4 were endogenously tagged with mCherry (Figure 1). Mei4 is required for a number of meiotic prophase events, including entry into meiosis I, after the pre-meiotic S-phase [41, 49]. Synchronised meiotic cell cultures, using *h*^*−*^*/h*^*−*^ *mat1-Pc pat1-114* diploid cells [50], showed expression of Mei4 in the early pre-meiotic S-phase (Supplementary Figure S1A and B). In live cell imaging, Mei4-mCherry became visible in the nucleus from the early meiotic prophase, as indicated by changes in signal intensity in the nucleus (Figure 1 and Supplementary Figure S1C: time point 0, defined as t0). Entry into meiosis I is marked by SPB separation, which we define as time point t3 (Figure 1). During the meiotic prophase, telomeres are clustered near the SPB, representing the ‘bouquet’ (Figure 1b) [21, 28]. The oscillation of the SPB-chromosomes ceases prior to meiosis I, which we define as time point t1 (Figure 1 and Supplementary Figure S1D). The time point for bouquet termination (t2) was defined as dispersion and dissociation of Taz1-YFP foci from the SPB (Figure 1 and Supplementary Figure S2). Thus, by assuming Mei4 nuclear staining becomes visible during the pre-meiotic S-phase, the duration of meiotic prophase is defined as the time period from t0 to t3 (calculated as t3–t0) in this system. Meiotic prophase can be further divided into the horsetail stage (t1–t0) and the post-horsetail stage (t3–t1) (Figure 1a).

Figure 1e shows individual value plots from which one can deduce quantitative measures of meiotic progression. Two-thirds of wild-type cells separated their SPBs and underwent meiosis I within 2 h of Mei4 expression. This is consistent with the results of a previous study defining the duration of meiotic prophase using the end of karyogamy as a starting point [26].

To better understand the regulation of bouquet maintenance throughout meiotic prophase, we deleted genes that are involved in meiotic recombination but not essential for completion of DNA double-strand break (DSB) repair. Rdh54 is a meiotic recombination-specific Rad54-like DNA translocase. In fission yeast, deletion of *rdh54*^+^ leads to modest defects in homologous recombination and minor effects in the progression of meiotic prophase [51]. Our live cell imaging confirmed the delay in meiotic prophase exit, and found that the bouquet stage was extended in the *rdh54*Δ strain (Figure 1c and e). At the end of meiotic prophase, *rdh54*Δ cells behaved similarly to wild-type cells: SPB separation and telomere release from the SPB occurred simultaneously regardless of the duration of meiotic prophase (Figure 1e and Supplementary Figure S3A). Furthermore, no defects associated with SPB separation were observed (Supplementary Figure S4). Similar results were obtained by deletion of *meu13*^+^, which encodes the orthologue of budding yeast Hop2p and is involved in meiotic recombination and homologous chromosome pairing [52] (Figure 1e and Supplementary Figure S5). Thus, the length of the bouquet stage is extended in two different meiotic recombination mutants, but the timing of telomere release from the bouquet remains tightly controlled.

**Figure 2:**
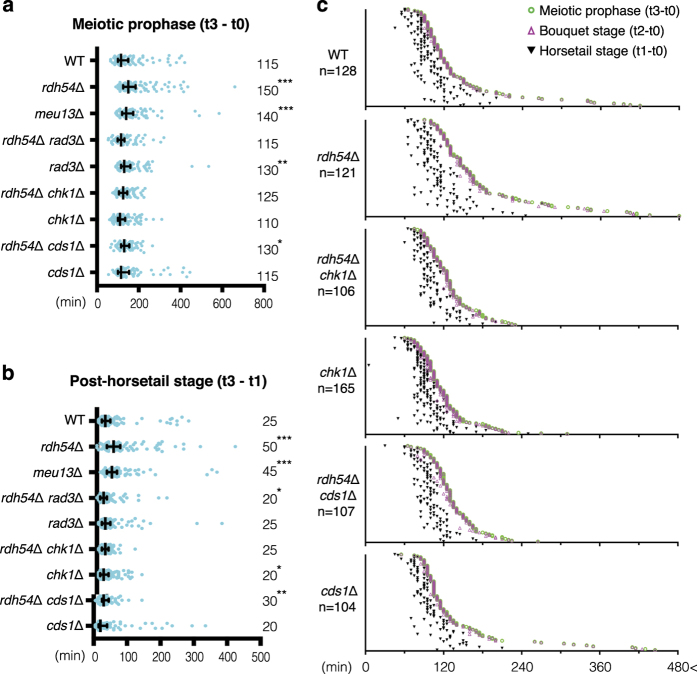
Rad3/ATR-dependent extension of the bouquet stage. (**a**, **b**) Distribution graphs calculated from dot plots in Figures 1e and 2c. Median durations are indicated on the right. The outside bars represent interquartile range. Significant differences over wild type are indicated as asterisks (the Mann–Whitney nonparametric test: * at *P*<0.05, ** at *P*<0.01 and *** at *P*<0.001). (**a**) Duration of meiotic prophase [t3–t0]. (**b**) Duration of the post-horsetail stage, which represents the time from SPB settling until entry into meiosis I [t3–t1]. (**c**) Individual dot plots of meiotic prophase time course in the DNA damage checkpoint mutants. Data for WT and *rdh54*Δ are taken from Figure 1e. See the graph in Figure 1e for details. For values in each sample, see Supplementary Table S2.

Although meiotic prophase (t3–t0) was significantly extended in *rdh54*Δ and *meu13*Δ cells (Figure 2a), the duration of the horsetail stage (t1–t0) was only mildly extended compared with wild type (Supplementary Figure S3B). Interestingly, the post-horsetail stage, the time between the SPB settling and its separation (t3–t1), was on average doubled in the *rdh54*Δ and *meu13*Δ mutants compared with wild type (Figure 2b). Kymograph analysis of nuclear movement in wild-type cells showed settling of the SPB and telomeres at the centre of the cell prior to SPB separation (Supplementary Figure S6) [34]. In *rdh54*Δ cells, this settling period of the SPB and telomeres was significantly extended (Supplementary Figures S1D and S6). Thus, the bouquet stage, especially the post-horsetail stage, is prolonged in the meiotic recombination mutants.

### Rad3–Chk1 dependent extension of the post-horsetail stage in the recombination mutants

A previous study showed that extension of meiotic prophase in the absence of Meu13 is dependent on Rad3 [48]. Indeed, extension of meiotic prophase was not observed in *rdh54*Δ *rad3*Δ double mutant cells (Figure 2a). We found that deletion of *rad3*^+^ abolished extension of the post-horsetail stage in *rdh54*Δ cells (Figures 1d, e and 2b). A similar result was obtained by deletion of *chk1*^+^ (Figure 2b and c), suggesting that Rad3 and Chk1 act together. Cds1, involved in DNA replication checkpoint and termination of chromosome oscillation, appears to play a more minor role, as the post-horsetail stage in *cds1*Δ *rdh54*Δ double mutant cells was partially extended compared with *cds1*Δ single mutant cells (Figure 2b and c). Chk1 stabilises the spindle assembly checkpoint protein Mad2 [53], and Mad2 appears at the SPB prior to SPB separation in fission yeast [54]. However, deletion of *mad2*^+^ did not abolish the extension of the post-horsetail stage in *rdh54*Δ meiosis (Supplementary Figure S7), suggesting that the Mad2-dependent pathway is dispensable from this process. Collectively, our data suggest that Rad3–Chk1 activation is the main DNA damage checkpoint pathway restraining meiotic prophase after the horsetail stage.

### Significant numbers of DNA damage foci from meiotic recombination are diminished during the post-horsetail stage

**Figure 3:**
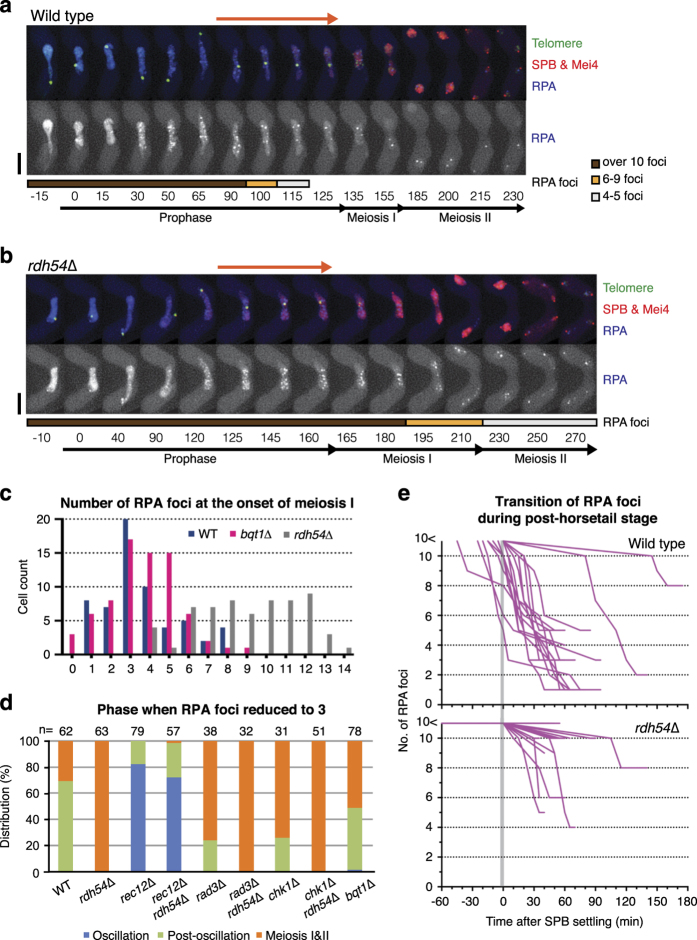
RPA foci diminishes before entry into meiosis I in wild type. (**a**, **b**) Series of frames from films of cells undergoing meiosis. The SPB, Mei4, telomeres and the RPA component Ssb2 were visualised *via* endogenously tagged Sid4-mCherry, Mei4-mCherry, Taz1-YFP and Ssb2-Cerulean. RPA foci represent sites of chromosome replication and DNA damage (separate channel is shown below merged image). Cell images were captured every 5 min, and selected time frames are shown. Numbers below the slides represent minutes after Mei4 staining became visible in the nuclei. The post-horsetail stage is highlighted with the orange arrow lines. The coloured bar below the RPA row indicates the number of RPA foci: over 10 foci (brown), 6–9 foci (orange) and 4–5 foci (light grey). Scale bars equal 5 μm. (**a**) An example image of a wild-type cell experiencing a long post-horsetail stage (45 min, highlighted above the frames) is shown. The number of distinct foci of RPA decreased when the SPB decelerates (85 min), and further decreased prior to meiosis I. Two RPA foci remained through meiosis. (**b**) *rdh54*Δ single mutant cells maintain more than 10 distinct foci of RPA throughout meiotic prophase and the number is reduced through meiosis. However, a few strong RPA foci were retained after meiosis. Sixty-three cells were examined and most of the cells retained 4–6 strong RPA foci at the end of meiosis. (**c**) Graph showing a number of RPA foci at entry into meiosis. Most of cells enter meiosis when RPA foci decreased to 3–4 foci in both wild type and *bqt1*Δ cells. Examined sample numbers are WT (*n*=60), *bqt1*Δ (*n*=74) and *rdh54*Δ (*n*=62). (**d**) Distribution graph showing the stage of meiosis where RPA foci are largely diminished. The sample number (*n*=) is showing above. (**e**) Graphs showing transition of the number of RPA foci through the post-horsetail stage. Eighteen wild-type cells that exhibited a prolonged post-horsetail stage (Top) and 18 *rdh54*Δ cells (Bottom) were selected and their RPA foci were counted and plotted until entry into meiosis I. The *y*-axis indicates a number of RPA foci. The *x*-axis indicates time after SPB settling (the post-horsetail stage). Most cells harbour more than 10 RPA foci during the horsetail stage (minus values of *x*-axis).

Our single-cell analysis revealed that extension of the post-horsetail stage was also observed in some wild-type cells (Figure 2b and c and the example is shown in Figure 3a). To assess whether this was associated with delayed DNA repair, we visualised the recombination protein Rad52, a cytological marker of on-going homologous recombination repair, and replication protein A (RPA), a marker for DNA replication and recombination intermediates. In wild-type cells, Rad52 stained the nucleus but the intensity diminished prior to SPB separation (Supplementary Figure S8A). Clear foci were detected when RPA was visualised (Figure 3). Numerous foci of RPA were observed during the horsetail stage, and the number of foci reduced when chromosome movement ceased (Figure 3a). However, a few RPA foci could still be observed during meiosis in wild-type cells. This phenomenon has been reported previously using the Rad51 DNA DSB marker [55]. The median value of RPA foci detected at the onset of meiosis I was 3 in wild type (Figure 3c and d). In *rec12*Δ cells, which lack the meiotic recombination process, RPA foci diminished during the early horsetail stage (Figure 3d and Supplementary Figure S9A). Although some *rec12*Δ cells retained 1–2 RPA foci throughout meiosis, the post-horsetail stage was not extended (Supplementary Figure S9A and C). A previous report showed that DNA damage occurring during DNA replication does not arrest meiotic progression [55]. Hence, the few remaining RPA foci observed in wild-type cells are likely to have originated from the pre-meiotic S-phase. Importantly, the post-horsetail stage was prolonged while greater numbers of punctate RPA foci were detected (Figure 3e). This suggests that the bouquet configuration is retained without oscillation when DNA repair during meiotic recombination is delayed in wild-type cells. Thus, our results suggest that telomere release is suppressed under impaired meiotic recombination.

In *rdh54*Δ cells, distinct Rad52 and RPA foci were observed but gradually reduced through meiotic prophase (Supplementary Figure S8B and Figure 3b, respectively). Although the post-horsetail stage was prolonged, the cells eventually entered meiosis with significant numbers of RPA foci remaining (Figure 3c and d). Nevertheless, through two rounds of meiotic segregation, the number of RPA foci was further reduced (Figure 3b). The number of residual RPA foci and the extension of the post-horsetail stage in *rdh54*Δ cells were diminished in the absence of Rec12, indicating that unrepaired DNA during meiotic recombination extends the post-horsetail stage (Figure 3d and Supplementary Figure S9B and C). Although deletion of *rad3* and *chk1* diminished extension of the post-horsetail stage in *rdh54*Δ cells, this was not due to completion of meiotic recombination, as a significant number of RPA foci were detected throughout meiosis in *rad3*Δ *rdh54*Δ and *chk1*Δ *rdh54*Δ double mutants (Figure 3d and Supplementary Figure S10A–D). Such forced entry into the chromosome segregation phase in the absence of the DNA damage checkpoint led to impaired chromosome segregation (Supplementary Figure S10E and F). In conclusion, residual RPA foci activate the Rad3–Chk1 pathway, which restricts the post-horsetail bouquet stage. The functional significance of this stage remains unknown. Therefore, we further explored the role of the bouquet after the end of chromosome oscillation.

### Bouquet formation ensures entry into meiosis I

**Figure 4:**
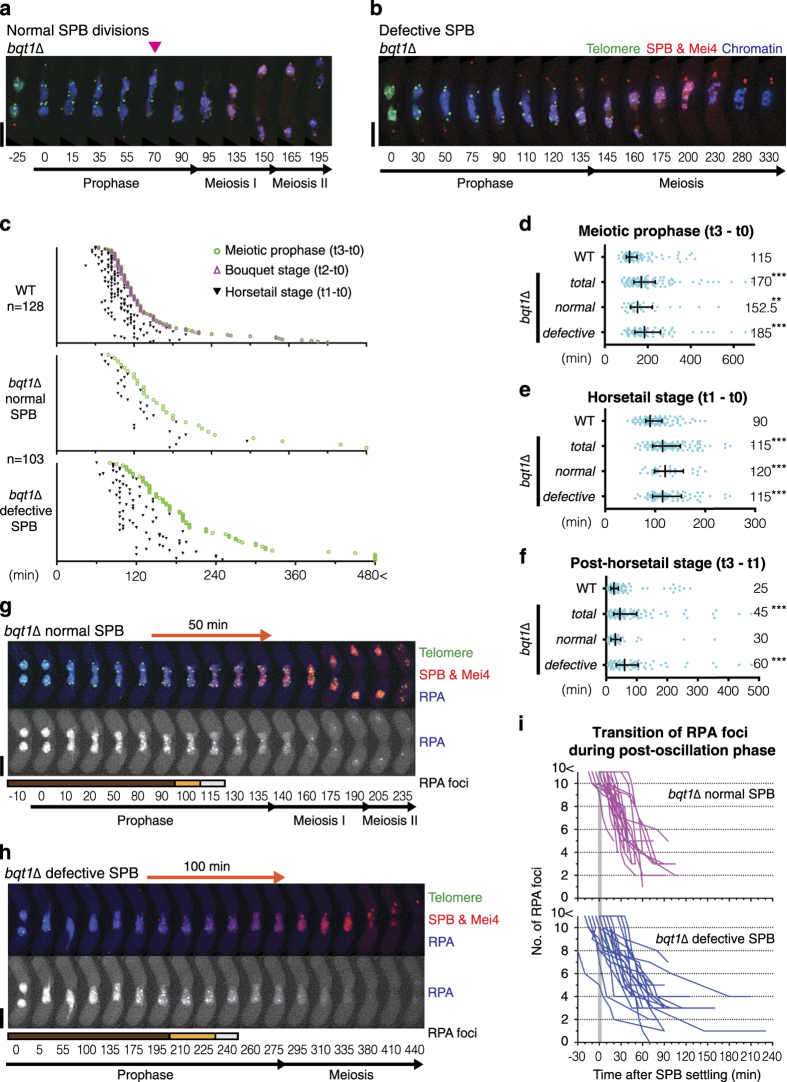
Meiotic prophase is prolonged in the absence of Bqt1. (**a**, **b**, **g** and **h**) Series of frames from a film of *bqt1*Δ cells undergoing meiosis. Cell images were captured every 5 min, and selected time frames are shown. Numbers below the slides represent minutes after Mei4 staining became visible in the nuclei (t0). Telomeres, the SPB and Mei4 were visualised *via* endogenously tagged Taz1-YFP, Sid4-mCherry and Mei4-mCherry, respectively. Chromosomes and RPA were visualised by Hht1-Cerulean (**a**, **b**) and by Ssb2-Cerulean (**g**, **h**), respectively. Scale bars=5 μm. (**a**) An example of a *bqt1*Δ cell that successfully underwent meiosis is shown. During meiotic prophase, telomeres form foci but do not associate with the SPB in *bqt1*Δ cells. The SPB moves back and forth without chromosomes during the horsetail stage. In these cells, a chromosome is eventually captured during the SPB movement (at the 70 min time point, highlighted by a pink arrowhead). Chromosome condensation and the SPB separation are observed at the 95th minute time point. The separated SPB successively segregates chromosomes through meiosis I and II. (**b**) An example of a *bqt1*Δ cell that exhibits an aberrant SPB is shown. Although the SPBs do not separate, it duplicates and chromosome condensation is observed at the 135 min time point, which represents entry into meiosis. Dynamic rearrangement of chromosomes without the SPB is observed through meiosis, and the SPB eventually becomes fragmented (at the 330 min time point). (**c**) Individual dot plots of meiotic prophase time course in *bqt1*Δ cells categorised by the SPB phenotypes. The sample number (*n*=) is indicated below the genotype. Among *bqt1*Δ cells, cells exhibited normal and defective SPB are 30 and 73, respectively, in this study. Data for WT is taken from Figure 1e. See the graph in Figure 1e for details. Proportion of the time distributions is reminiscent of that in *rdh54*Δ cells (Figure 1e). For values in each sample, see Supplementary Table S2. (**d**–**f**) Distribution graphs calculated from dot plots. Median durations are indicated on the right. The outside bars represent interquartile range. Statistically significant differences over wild type are indicated as asterisks (the Mann–Whitney nonparametric test: ** at *P*<0.01 and *** at *P*<0.001). Data from *bqt1*Δ cells are phenotypically categorised into normal SPB and defective SPB. Data for WT is taken from Figure 2a and b and Supplementary Figure S3b. (**d**) Duration of meiotic prophase (t3–t0). (**e**) Duration of the horsetail stage from Mei4 expression to SPB settling (t1–t0). (**f**) Duration of the post-horsetail stage, which represents the length of time from SPB settling until entry into meiosis I (t3–t1). (**g**, **h**) Remaining RPA foci during the post-horsetail stage (or SPB settling period) in *bqt1*Δ cells. The duration of the post-horsetail stage is highlighted with the orange arrow lines. Coloured bars below the RPA row indicate the number of RPA foci: over 10 foci (brown), 6–9 foci (orange) and 4–5 foci (light grey). *bqt1*Δ cells that complete meiosis (**g**) have diminished RPA foci before meiosis I. Cells that exhibit aberrant SPBs (**h**) experienced a long post-horsetail stage even though RPA foci are diminished. (**i**) The graph represents the transition of the number of RPA foci through the post-horsetail stage in *bqt1*Δ cells. See details of the graph in Figure 3e. Twenty-four of each *bqt1*Δ cell type, which exhibited normal or defective SPBs, were analysed and plotted until entry into meiosis I.

Retention of bouquet formation throughout meiotic prophase prompted us to investigate a possible role for the bouquet in exit from meiotic prophase. To this end, meiotic progression was monitored in *bqt1*Δ cells. We found that elimination of Bqt1 conferred extension of meiotic prophase (Figure 4a–d). In *bqt1*Δ cells, approximately half of the cell population undergoes successful meiosis due to random contacts between the SPB and the centromere (Figure 4a) [34]. The SPB oscillation period, which represents ‘the horsetail stage’, was extended in the entire population of *bqt1*Δ cells (Figure 4c and e). The post-horsetail stage was not significantly extended in *bqt1*Δ cells that successfully separated the SPB (Figure 4f). However, we observed significant extension of the post-horsetail stage in cells with defective meiotic SPBs (Figure 4f). Thus, our data suggest that the bouquet configuration of chromosomes contributes to the progression of events during meiotic prophase *via* the SPB. Failure to connect chromosomes with the SPB leads to severe extension of the post-horsetail stage.

Bouquet formation is crucial for facilitating pairing and recombination of homologous alleles and excluding ectopic recombination. Thus, we hypothesised that loss of the bouquet and homologous pairing might impair the efficiency of DNA DSB repair, thereby extending meiotic prophase. Accordingly, we found that extension of the horsetail stage in *bqt1*Δ cells was partly associated with retention of RPA foci (Figure 4g–i). However, RPA foci were largely diminished during the post-horsetail stage (Figures 3c, d and 4i), suggesting that DNA DSBs were repaired during meiotic prophase in *bqt1*Δ cells.

**Figure 5:**
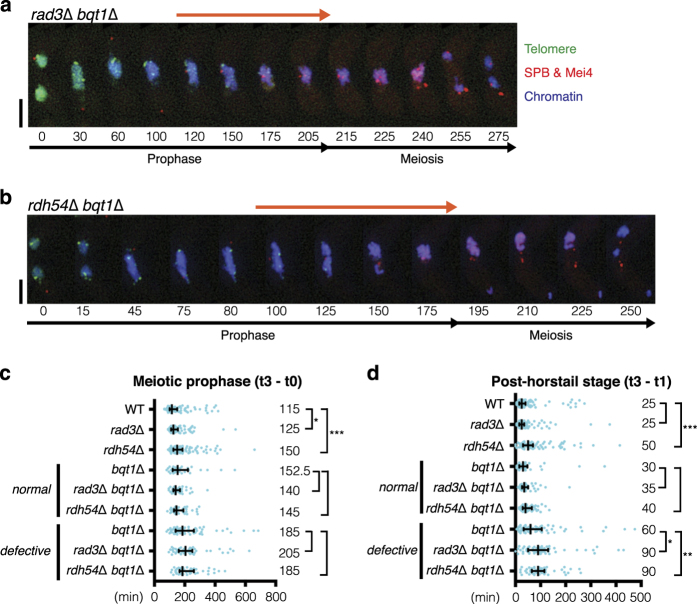
DNA damage checkpoint and repair independent extension of the post-horsetail stage in *bqt1*Δ. (**a**, **b**) Series of frames from a film of cells undergoing meiosis (**a**: *rad3*Δ *bqt1*Δ, **b**: *rad3*Δ *bqt1*Δ). Cell images were captured every 5 min, and selected time frames are shown. Numbers below the slides represent minutes after Mei4 staining became visible in the nuclei (t0). The post-horsetail stage is highlighted with the orange arrow lines. SPB, Mei4, telomeres and chromosomes were visualised *via* endogenously tagged Sid4-mCherry, Mei4-mCherry, Taz1-YFP and Hht1-Cerulean, respectively. Scale bars equal 5 μm. (**c**, **d**) Distribution graphs for (**c**) duration of meiotic prophase (t3–t0) and (**d**) duration of the post-horsetail stage (t3–t1). Median durations are indicated on the right. The outside bars represent interquartile range. Statistically significant differences between single and double mutants are indicated as asterisks (the Mann–Whitney nonparametric test: * at *P*<0.05, ** at *P*<0.01 and *** at *P*<0.001). Data from *bqt1*Δ cells are phenotypically categorised into normal SPB and defective SPB. Data for WT, *rad3*Δ and *rdh54*Δ and data for *bqt1*Δ are taken from Figures 2a,b and 4d,f. The sample number of the double mutants are; *rad3*Δ *bqt1*Δ *normal* (*n*=44), *rdh54*Δ *bqt1*Δ *normal* (*n*=54), *rad3*Δ *bqt1*Δ *defective* (*n*=55) and *rdh54*Δ *bqt1*Δ *defective* (*n*=55).

Whereas SPB-defective *bqt1*Δ cells extended the post-horsetail stage, RPA foci diminished at a similar rate to *bqt1*Δ cells with normal SPB function (Figure 4i). Curiously, deletion of *rad3* did not suppress extension of the post-horsetail stage in *bqt1*Δ cells and instead synergistically extended it (Figure 5). This implies that Rad3 becomes crucial for meiotic progression in the absence of the bouquet configuration. Severe extension of the post-horsetail stage was also observed in *rdh54*Δ *bqt1*Δ double mutants. Like the *bqt1*Δ single mutants, half of the cells with *rad3*Δ or *rdh54*Δ backgrounds exhibited SPB defects (Supplementary Figure S4). Importantly, *bqt1*Δ cells with functional SPBs did not extend the post-horsetail stage and, in these cells, synergistic extension was not observed. Thus, defective meiotic recombination is not a prerequisite for the extension of meiotic prophase observed in *bqt1*Δ cells that display the SPB defects. Our data therefore suggest that chromosome contact with the SPB promotes exit from the post-horsetail stage.

### The telomere bouquet stabilises CDK1^cyclin B^ at the SPB prior to its termination

**Figure 6:**
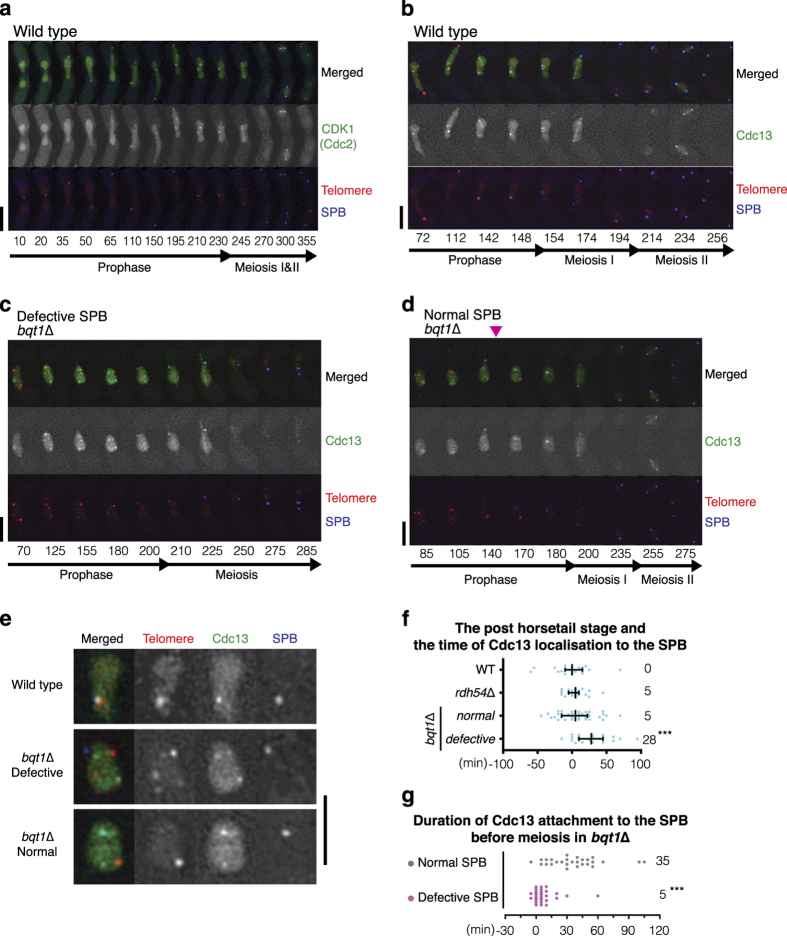
CDK1^cyclin B^ localises at telomeres during meiotic prophase. (**a**–**d**) Series of frames from films of wild type (**a**, **b**) and *bqt1*Δ (**c**, **d**) cells endogenously expressing Cdc2-YFP (**a**) or Cdc13-YFP (**b**–**d**), a CDK1-cyclin B marker. Telomeres and the SPB were visualised *via* endogenously tagged Taz1-mCherry and Sid4-Cerulean, respectively. Cell images were captured every 2 min for (**b**) and 5 min for (**a**, **c** and **d**), and selected time frames are shown. Numbers below the slides represent minutes from the beginning of the film. Scale bars=5 μm. (**a**) Localisation of CDK1 (Cdc2) throughout meiosis in wild type. (**b**) Localisation of CDK1^cdc13^ throughout meiosis in wild type. CDK1^cdc13^ spreads throughout the entire nucleus including where the telomeres are positioned and punctuated CDK1^cdc13^ foci are observed throughout meiotic prophase. A weak CDK1^cdc13^ focus starts to appear at the SPB late in the horsetail stage, and CDK1^cdc13^ foci switch from nucleoplasm to the SPB and telomeres at the end of the pre-meiotic phase. Once CDK1^cdc13^ has accumulated, telomeres are released and SPB separation commences. CDK1^cdc13^ spreads onto the formed spindle until metaphase. Twenty-two wild-type cells were analysed and all exhibited similar CDK1^cdc13^ behaviour. (**c**) In *bqt1*Δ meiosis, some CDK1^cdc13^ foci stably adjust their localisation to some telomere foci, but not at the SPB (until 180 min). Nucleoplasmic CDK1^cdc13^ foci diffuse at the end of meiotic prophase when telomeres disperse (telomere foci resolve) (200 min). The SPB gradually accumulates CDK1^cdc13^ while microtubules (CDK1^cdc13^ filaments) appears from nuclei. CDK1^cdc13^ is eventually degraded and the SPB becomes aggregated and fragmented. (**d**) Among the *bqt1*Δ mutants, cells undergoing successful SPB divisions showed accumulation of CDK1^cdc13^ foci at the SPB during meiotic prophase. In this example, the SPB captured a punctuated CDK1^cdc13^ focus during the horsetail stage at the 140 min time point, highlighted by a pink arrowhead, where it was retained until entry into meiosis. Such SPBs successfully underwent two subsequent divisions throughout meiosis. CDK1^cdc13^ relocates once the SPB captures the CDK1^cdc13^ signal. (**e**) The nuclei images at the end of meiotic prophase (Top: **b**, 148 min; Middle: **c**, 180 min; Bottom: **d**, 170 min) are enlarged. (**f**) Distribution graph showing the timing of CDK1^cdc13^ localisation at SPB after the horsetail stage in wild type (*n*=24), *rdh54*Δ (*n*=26) and *bqt1*Δ (*n*=61) cells. The *bqt1*Δ cells were categorised according to their SPB phenotypes: normal SPB (*n*=37) and defective SPB (*n*=24). The *x*-axis indicates time after SPB settling. Median is indicated on the right. The outside bars represent interquartile range. Significant differences over wild type are indicated as asterisks (the Mann–Whitney nonparametric test: *** at *P*<0.001). (**g**) Distribution graph showing the duration of CDK1^cdc13^ foci at SPB prior to meiotic entry in the *bqt1*Δ cells. Median is indicated on the right. The outside bars represent interquartile range. A total of 58 *bqt1*Δ cells were analysed and categorised according to their SPB phenotypes. Among those, 31 cells exhibited defective SPBs and failed to stabilise CDK1^cdc13^ at the SPB before meiosis (statistical significance from ‘normal SPB’ at *P*<0.0001, the Mann–Whitney nonparametric test).

We have shown that if meiotic recombination is delayed, the post-horsetail stage is extended *via* the Rad3–Chk1 pathway. A similar extension was observed in *bqt1*Δ cells but this delay was still observed in the *rad3*Δ background, suggesting a distinct mechanism. The Rad3–Chk1 pathway suppresses activation of CDK1 [45], which is required for SPB separation [40, 43]. To investigate how meiotic telomeres control exit from meiotic prophase, we monitored localisation of the CDK1 (Cdc2) and cyclin B (Cdc13) complex in *bqt1*Δ cells. It has been reported that the CDK1^Cdc13^ complex accumulates at centromeres before meiosis begins, then relocates to the SPB and spreads throughout the spindle during meiosis [56]. Using telomere and SPB markers along with Cdc2-YFP, we observed distinct nucleoplasmic foci that colocalise with telomeres and the SPB throughout meiotic prophase (Figure 6a). Although Cdc2 interacts with a number of cyclins, Cdc13 is the only cyclin required for entering the chromosome segregation phases [40]. Cdc13-YFP showed distinct nucleoplasmic foci during the horsetail stage (Figure 6b). A Cdc13 focus began to appear at the SPB once the SPB oscillation had ceased. Prior to the chromosome segregation phase, the nucleoplasmic Cdc13 foci diminished and a bright single focus appeared at the SPB and telomeres (148 min time point; enlarged nuclear images are shown in Figure 6e). Telomeres were then released from the SPB, followed by SPB separation and spindle formation. In meiosis I, CDK1^Cdc13^ remained at the SPB, rather than at telomeres, and then spread throughout the formed meiotic spindle. The fact that both wild type and *rdh54*Δ cells displayed colocalisation of one bright Cdc13 focus with telomeres and the SPB during late prophase, especially at the end of SPB oscillation (Figure 6f), suggests that pre-accumulation of Cdc13 at the SPB during the post-horsetail stage is unrelated to the presence of any remaining DNA damage.

In the *bqt1*Δ mutants, Cdc13 formed numerous foci within the nucleus and some of them localised to telomeres but not at the SPB during meiotic prophase (Figure 6c). The SPB, which was unable to accumulate Cdc13 at the later stages of meiotic prophase, failed to separate (Figure 6c). In these defective cells, Cdc13 focus appeared at the SPB during meiosis I when microtubules, represented by CDK1^Cdc13^ filaments, were formed from the nucleus (the 210 min time point). Subsequently, the unseparated SPB became fragmented (the 285 min time point). In contrast, the *bqt1*Δ mutant cells in which the SPB managed to stabilise Cdc13 during meiotic prophase (Figure 6d: pink arrowhead) underwent successful SPB separation, spindle formation and chromosome segregation. Overall, the *bqt1*Δ cells that exhibited impaired localisation of Cdc13 at the SPB during the post-horsetail stage showed SPB defects (Figure 6f), suggesting that the absence of Cdc13 at the SPB correlates with the delay in meiotic prophase exit in *bqt1*Δ. Accordingly, these cells failed to stabilise CDK1^Cdc13^ at the SPB before commitment to meiosis (Figure 6g). Thus, the telomere bouquet facilitates timely accumulation of CDK1^Cdc13^ at the SPB prior to its separation and spindle assembly.

### Premature termination of the bouquet leads to spindle defects

**Figure 7:**
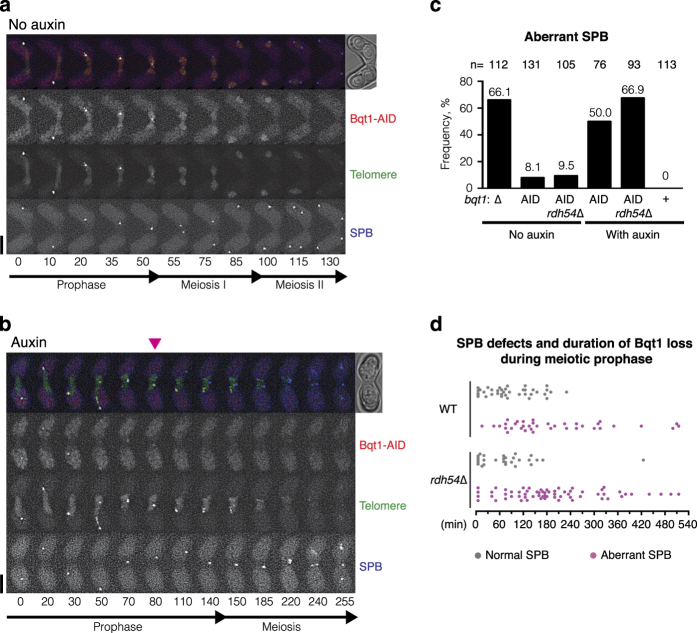
Loss of bouquet formation during meiotic prophase leads to aberrant SPB behaviour. (**a**, **b**) Series of frames from films (Supplementary Movies S1 and S2) of cells carrying AID-tagged Bqt1 and SCF^TIR1^ undergoing meiosis without (**a**) and with (**b**) auxin. Telomeres and the SPB were visualised *via* endogenously tagged Rap1-YFP (third panel) and Sid4-Cerulean (bottom panel), respectively. Cell images were captured every 5 min, and selected time frames are shown. Numbers below the slides represent minutes from the beginning of filming. Spore formation was photographed approximately 12 h after filming. Scale bars=5 μm. (**a**) Without auxin, Bqt1-AID foci (second panel) diminish when telomeres disperse and SPB divides. (**b**) Auxin-dependent loss of Bqt1-AID-mCherry signal (second panel) and premature termination of the bouquet, represented by dissociation of telomere foci from the SPB, were observed at the 70–80 min time point (arrowhead). Dissociated telomeres remained clustered and dispersed at the 150th minute time point. (**c**) Graph showing the frequency of dysfunctional SPBs observed with and without auxin and with and without AID tagging. Deletion of *bqt1*^+^ (Δ) is shown as a control. All other strains express SCF^TIR1^. The experiment was repeated in an *rdh54*Δ background. For auxin-induced Bqt1-AID destruction studies, only cells that exhibited loss of bouquet formation during meiotic prophase were counted (WT: *n*=76 out of 104, *rdh54*Δ: *n*=93 out of 105). Note that a small proportion of cells bearing Bqt1-AID exhibited bouquet defects even without auxin addition, implying that AID tagging can slightly destabilise Bqt1 in the presence of the SCF^TIR^ ubiquitin ligase. (**d**) Distribution graph of the length of time between Bqt1 loss and the onset of meiosis. Samples from (**c**) were categorised by SPB phenotypes; WT with normal SPB (*n*=38), WT with aberrant SPB (*n*=38), *rdh54*Δ with normal SPB (*n*=28), *rdh54*Δ with aberrant SPB (*n*=65). Grey and magenta dots indicate normal (functional) and defective SPBs at meiosis, respectively.

Bouquet formation and chromosome oscillation during early meiotic prophase facilitate homologous pairing [26, 29, 30], and our data suggest that bouquet formation during later meiotic prophase provides a foothold for CDK1^Cdc13^ to activate the SPB. To directly assess the requirement for telomere attachment to the SPB throughout meiotic prophase, we set up a system to conditionally terminate the bouquet stage. The AID-SCF^TIR1^ system is a widely-utilised method to selectively destroy target proteins [57, 58]. An auxin-inducible degron (AID) tag was fused to Bqt1 to disrupt bouquet formation by addition of auxin (Figure 7). When auxin was added before nitrogen starvation to induce meiosis, 28 out of 29 cells showed complete loss of telomere clustering and, like *bqt1*Δ cells, approximately half of these cells exhibited SPB and chromosome segregation defects, indicating that Bqt1-AID can be diminished by auxin addition (Supplementary Figure S11). In contrast, auxin activated SCF^TIR1^ did not impair meiosis of cells carrying Bqt1 without AID fusion (Figure 7c and Supplementary Figure S11). Without auxin, Bqt1-AID foci disappeared at the onset of meiosis I, and normal SPB segregation was observed (Figure 7a and Supplementary Movie S1). Collectively, our data showed that the E3 ubiquitin ligase complex SCF^TIR1^ can target AID-tagged proteins without affecting meiotic progression, and the bouquet can be efficiently disrupted by the addition of auxin.

To disrupt the bouquet in the middle of meiotic prophase, auxin was added 30 min before filming. Under this condition, cells showed auxin-dependent diffusion of Bqt1-AID foci during prophase, followed by telomere release from the SPB (Figure 7b). As a result, half of the cells exhibited aberrant SPBs at the end of meiosis (Figure 7c). In the example shown (Figure 7b and Supplementary Movie S2), loss of Bqt1 foci and disconnection of the SPB from telomeres were observed after the 80 min time point. The SPB alone moved back and forth and then settled at the centre. Telomere dispersion was observed at the 150-min time point, and finally the abnormal SPB became fragmented after the 240-min time point. In this auxin-inducible Bqt1 destruction system, the time duration between Bqt1 loss and the onset of meiosis varied between cells (Figure 7d). Cells that experienced a longer duration (over 3 h) of Bqt1 loss were likely to exhibit SPB defects. This is due to extension of meiotic prophase caused by loss of bouquet configuration (Figure 4d: median value 170 min). Visualisation of RPA foci confirmed that this premature termination of the bouquet occurred while numerous RPA foci remained (Supplementary Figure S12A and B). We did not observe any correlation between the length of bouquet formation and SPB deficiency (Supplementary Figure S12C). To summarise, our system demonstrated that destruction of Bqt1 during the meiotic recombination stage reproduced the phenotype and results observed in the *bqt1*Δ strain. Thus, bouquet maintenance throughout meiotic prophase is crucial for SPB function.

**Figure 8:**
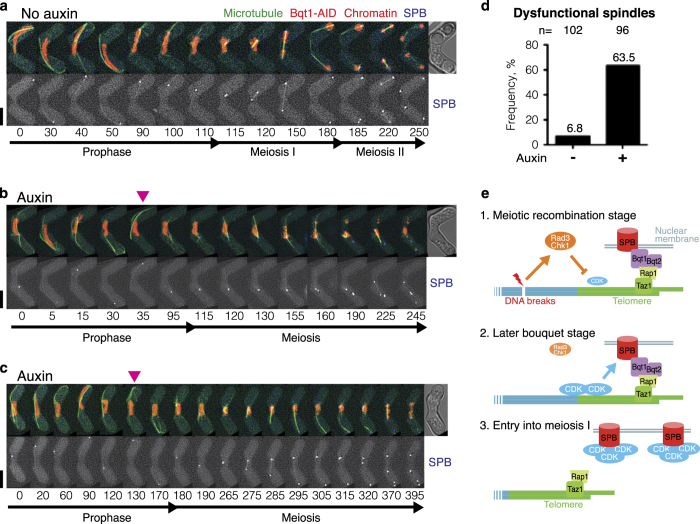
Bouquet formation throughout meiotic prophase is crucial for meiotic spindle formation. (**a**–**c**) Series of frames from films (Supplementary Movies S3, S4, S5, respectively) of *rdh54*Δ cells carrying Bqt1-AID and SCF^TIR1^ undergoing meiosis without (**a**) and with (**b**, **c**) auxin. Chromosomes, microtubules and the SPB were visualised *via* endogenously tagged Hht1-mCherry, GFP-Atb2 and Sid4-Cerulean (bottom panel), respectively. Cell images were captured every 5 min, and selected time frames are shown. Numbers below the slides represent minutes from the beginning of the film. Spore formation was photographed approximately 12 h after filming. Scale bars=5 μm. (**a**) An example of normal meiosis without auxin addition. Cytoskeleton microtubules promote the SPB and nuclear oscillation during meiotic prophase. Cytoplasmic microtubules depolymerise before spindle formation (at the 110 min time point). Bipolar spindles are established between divided SPBs. (**b**) Auxin-induced bouquet termination leads to monopolar spindle formation in meiosis I. In this example, disruption of the bouquet, represented by detachment of chromosomes from the SPB, is observed at the 35 min time point (arrowhead). Depolymerisation of cytoplasmic microtubules is observed at the 95 min time point, and a monopolar spindle is formed from a duplicated undivided SPB. One of the SPBs eventually dislodges and another SPB divides and establishes a bipolar spindle at the 225 min time point. (**c**) Auxin-induced bouquet termination leads to formation of a dysfunctional bipolar spindle (skipping metaphase). In this example, disruption of the bouquet is observed at the 120–130 min time points (arrowhead), followed by microtubule depolymerisation and chromosome condensation at the 180th minute time point. The spindle is not established until the 265 min time point. A monopolar spindle is initially formed and becomes bipolar. However, the established bipolar spindle does not capture chromosomes and immediately elongates and pushes one of the SPBs away. Another SPB, which contacted with chromosomes, establishes a second bipolar spindle at the 320 min time point. (**d**) Graph showing the frequency of dysfunctional spindles after premature termination of the bouquet stage initiated by addition of auxin. The sample number is indicated above (*n*=). (**e**) A model of the telomere checkpoint and spindle control. Our data indicate that chromosome contact with the SPB until a late stage of meiotic prophase is required for the formation of functional spindles. Completion of meiotic recombination is signalled from telomeres to the SPB (Sad1) *via* CDK to promote timely SPB separation. Therefore, the telomere bouquet synchronises recombination completion and SPB maturation for faithful meiotic progression. (1) During meiotic recombination, DNA breaks activate Rad3 and Chk1, which in turn suppress CDK-cyclin activity and retain bouquet formation. CDK-cyclin starts to localise near the telomeres. (2) Completion of the meiotic recombination stage terminates the Rad3–Chk1 checkpoint. CDK-cyclin accumulates at telomeres and the SPB. (3) Telomeres dissociate from the SPB and CDK initiates SPB separation and spindle formation.

To clarify whether impaired chromosome segregation is associated with spindle defects, microtubules were visualised (Figure 8a and Supplementary Movie S3). We utilised the AID system in *rdh54*Δ background cells due to their longer post-horsetail stage. As expected, failure to form a functional bipolar spindle was observed only in cells that lost Bqt1 during meiotic prophase (Figure 8d). Meiotic spindle phenotypes observed in the bouquet-deficient mutants included: monopolar spindles (shown in Figure 8b and Supplementary Movie S4), no spindle formation from the SPB (but microtubule formation from the nucleus) [28], and bipolar spindles that do not arrest at metaphase and immediately extend without capturing chromosomes (namely ‘skipping metaphase’ [28], shown in Figure 8c and Supplementary Movie S5). In the latter phenotype, the defective bipolar spindles are presumably caused by impaired centromere reassembly [35]. In conclusion, our data indicate that premature termination of the bouquet can cause spindle defects.

## Discussion

Both bouquet formation and chromosome oscillation promote homologous pairing. However, the bouquet configuration extends beyond the oscillation period. Our single-cell analysis along with the AID system revealed that bouquet formation plays a crucial role during later meiotic prophase. Residual DNA damage from meiotic recombination activates the DNA damage checkpoint proteins Rad3 and Chk1, which maintain bouquet formation and meiotic prophase beyond the horsetail stage. CDK1^Cdc13^ colocalises near to the telomeres prior to the termination of the bouquet stage and facilitates the timely activation of CDK1 for SPB separation and spindle formation. Thus, the DNA damage checkpoints monitor meiotic recombination to control the timing of CDK activation, and the telomere bouquet facilitates accumulation of CDK at the SPB upon onset of meiosis I (Figure 8e). Continuous formation of the bouquet throughout meiotic prophase is therefore crucial for meiotic progression and faithful chromosome segregation in fission yeast. These findings illuminate a crucial function of the bouquet in coordinating the timing of meiotic spindle maturation with the end of the meiotic recombination stage in fission yeast meiosis.

SPB separation requires accelerated activation of CDK1 *via* positive feedback loops [37, 43, 59]. Our live cell imaging in bouquet mutants showed that CDK1^Cdc13^ can localise near to the telomeres and therefore to the SPB during the later bouquet stage. The delayed exit from meiotic prophase and aberrant SPBs observed in *bqt1*Δ cells were associated with delayed accumulation of CDK1^Cdc13^ at the SPB. This is presumably one of the reasons why unintentional contact of the SPB with a centromere, which accommodates CDK1^Cdc13^ during meiotic prophase, rescues the spindle defects in the bouquet mutants (Figure 6d) [34]. Therefore, heterochromatic regions of chromosomes act as a platform for CDK, and pre-concerted recruitment of CDK1^Cdc13^ *via* bouquet formation is likely critical for initiation of a positive feedback loop and timely activation of CDK1 at the onset of meiosis. Hence, we propose that the bouquet regulates spatial activation of CDK and is dispensable for the activity of DNA damage response factors.

The inner nuclear membrane protein Sad1 appears to be crucial for the initiation of local membrane breakdown, *fenestration*, in both mitosis and meiosis [33, 60]. It remains to be established how CDK localises to the chromosome and the SPB. Nevertheless, bouquet-dependent prior recruitment of CDK and resultant activation of Sad1 may permit reconstruction of the nuclear membrane to promote proper bipolar spindle formation. We have previously reported numerous phospho-modifications of a telomeric protein, Rap1, occurring throughout meiotic prophase [61]. Hence, meiotic telomeres are likely to accommodate not only CDK but also other kinases and signalling factors, which might be required for CDK activation and functional nuclear membranes and SPBs. Further investigation of meiotic telomeres and the meiotic LINC complex is anticipated to elucidate how the association of telomeres with LINC promotes meiotic progression.

Our single-cell analysis of fission yeast *bqt1*Δ cells suggests that the bouquet configuration facilitates progression of both horsetail and post-horsetail stages. Specifically, SPB defects in the absence of Bqt1 were associated with a prolonged post-horsetail stage, which was associated with impaired CDK1^Cdc13^ localisation to the SPB. Previous studies using a *pat1-114* synchronised meiosis culture suggested that DNA breaks in *bqt1*Δ meiosis are largely repaired on time, and do not document a significant delay in meiotic progression [24]. However, without activation of the pheromone pathway in *pat1-114* meiosis, centromere association with the SPB is retained during meiotic prophase [50]. This implies that the bouquet defect in *bqt1*Δ cells was bypassed by centromere attachment to the SPB. In the previous study, elimination of the telomere binding protein Taz1 did not significantly alter the duration of meiotic prophase [26]. This is partly due to retention of Rap1 on telomeres *via* another telomeric protein, Poz1 [38, 62], which reduces the frequency of SPB defects [28]. *taz1*Δ also impairs telomere integrity and induces telomere damage and rearrangements, [63, 64] which leads to increased homologous recombination at the telomere-proximal regions [28]. Thus, while the DNA repair process is intact, termination of meiotic prophase is delayed in bouquet-deficient meiosis.

Studies of DNA damage checkpoint mutants suggest that Rad3 monitors the whole process of meiotic prophase in fission yeast (this work and [44, 46, 51]). Rad3 activates Cds1 and Mek1 in early prophase followed by Chk1 [48]. Whereas Mek1 is the major pathway controlling extension of meiotic prophase [47], its expression is restricted to early meiotic prophase [48]. Our assays suggest that *chk1*Δ largely diminishes extension of the post-horsetail stage observed in *rdh54*Δ cells. Therefore, it is likely that both suppression of Cdc25 and activation of Wee1 are required for arrest in meiotic prophase. As Chk1 is activated later than Mek1, we speculate that Chk1 monitors persistent meiotic recombination before exit from meiotic prophase and extends the post-horsetail stage by activating Wee1.

In most organisms, completion of DNA repair and chromosome synapsis is monitored by the pachytene checkpoint to prevent precocious entry into meiosis I [19, 20]. Because fission yeasts do not have the synaptonemal complex, cells skip the pachytene stage and enter meiosis immediately after the meiotic recombination stage. The presence of a pachytene-like checkpoint has never before been reported in fission yeast. We propose that ATR-Chk1 acts as a monitor of termination of the meiotic recombination stage. The telomere bouquet operates a downstream signalling pathway to control activation of CDK and reconstruction of the nuclear membrane *via* the LINC complex for subsequent meiosis. Since telomere structure and the mechanism of bouquet formation are well conserved between fission yeast and mammals [4, 14, 62, 65], we speculate that this bouquet-associated meiotic control could be conserved to mammals, perhaps together with the ‘canonical’ pachytene checkpoint associated with chromosomal synapsis. In mouse spermatogenesis, the bouquet stage can be extended in mutants of the regulators for meiotic recombination, such as *atm* and *mlh1* [66–68]. Unsuccessful bouquet formation in spermatogenesis leads to permanent prophase arrest at the pachytene-like stage, or apoptosis [8, 10, 11, 13, 14]. This could be due to impaired spatial and temporal regulation of CDK activity in the absence of the bouquet configuration. Our findings shed light on the presence of a meiotic regulatory mechanism that synchronises chromosomal and spindle dynamics, and pave the way for understanding the functions of the telomere bouquet.

## Materials and Methods

### Strains and media

The genotypes of the strains used for this study are listed in Supplementary Table S1. All media and supplements were purchased from Formedium (Swaffham, UK). Fission yeast was grown at 32 °C in standard YES media. Mating and meiosis were induced at 26 °C in EMM lacking nitrogen unless otherwise indicated.

### Strain construction

A strain exogenously expressing Cdc13-YFP was reported before [56]. A strain exogenously expressing Ssb2-Cerulean was described before [69]. For tubulin visualisation, GFP was inserted at the start codon of the endogenous *atb2*^+^ [70]. Mei4 tagged strains were generated by insertion of mCherry or nine tandem PK (or V5) epitope tag (9xPK) coding genes before the stop codon of the *mei4*^+^ gene. A strain exogenously expressing Cdc2-YFP was generated by insertion of the YFP coding gene before the stop codon of the *cdc2*^*+*^ gene. To fuse the AID tag to Bqt1, the three-tandem PK epitope tags, AID and mCherry coding genes and a kanMX6 cassette were successively inserted to express the Bqt1-3xPK-AID-mCherry fusion protein, and the endogenous promoter was replaced with the thiamine repressible *nmt81* promoter. Other protein markers were endogenously tagged at the C-terminus as previously described [28, 61, 71]. *rdh54*^+^, *meu13*^+^ and *cds1*^+^ were deleted by replacement with the zeocin resistance cassette zeoCV (CMV-zeo) using a standard PCR-based gene targeting method described before [71]. Transformants carrying the zeoCV cassette were selected on YES media containing 100 μg ml^−1^ Zeocin (InvivoGen, San Diego, CA, USA).

### Synchronised meiosis, western blot and FACS analysis

Details were described previously [61]. Briefly, a logarithmically growing *pat1-114* diploid strain carrying Mei4-9xPK was transferred to EMM media lacking a nitrogen source (EMM-N), and was incubated for another 15–16 h to arrest cells in the G1 phase at 26 °C. To inactivate the *pat1* kinase gene and induce meiosis, the temperature was shifted to 34 °C, cultures were supplemented with one-fifth volume of EMM media pre-warmed to 34 °C and meiotic fractions were collected at the required time points. Each fraction was subjected to DNA content analysis by FACS using the Muse Cell Cycle Assay Kit (Merck Millipore, Park Watford Hertfordshire, UK) and protein extraction using 20% trichloroacetic acid. Mei4-PK, Cdc2 and Cdc13 were detected using 1/4 000 anti-V5 (PK) (Bio-Rad, Watford Hertfordshire, UK), 1/5 000 anti-PSTAIRE peptide and 1/1 000 anti-Cdc13 (Santa Cruz Biotechnology, Dallas, TX, USA) antibodies, respectively.

### Microscope image acquisition

As previously described [61], imaging was carried out with a DeltaVision Elite (Applied Precision, Buckinghamshire, UK) comprising an Olympus IX71 inverted fluorescent microscope, and Olympus UPlanSApo ×100, NA 1.40, oil immersion objective and a CoolSNAP HQ2 camera cooled to −30 °C (Roper Scientific, Sarasota, FL, USA). Cells were adhered to 35 mm glass culture dishes (MatTek, Ashland, MA, USA) precoated with 0.2 mg ml^−1^ soybean lectin (Calbiochem, Merck Millipore) and immersed in EMM-N media. Culture dishes were placed on the inverted microscope stage in an Environmental Chamber at 28 °C.

For live cell imaging, mCherry, YFP (or GFP) and Cerulean signals were captured with 1.2 s (32% filter), 1.5 s (32% filter) and 0.5 s (32% filter) exposures using Optical Axis Integration, which acquires 3.6 μm of *z*-axis by a continuous z sweep. This was repeated every 300 s for approximately 12 h. Images were deconvolved and analysed using SoftWoRx 5.5 (Applied Precision). Dead cells observed during the imaging and the subjects moving out of focus were excluded from the study.

### Auxin-inducible system

Freshly overnight growing AID-optimised cells on EMM plates at 36 °C were inoculated into EMM-N media and incubated at 26 °C. After 3.5 h, thiamine was added to a final concentration of 5 μg ml^−1^ to reduce BQT1 mRNA expression level. After a further 1 h, cells were adhered to a MatTek glass bottom dish with EMM-N media containing thiamine and 185 μg ml^−1^ auxin, and the dish was set up on the microscope stage. To observe disruption of the bouquet in the middle of meiotic prophase, filming of zygotes commenced 30 min after auxin addition unless otherwise stated.
