# Supplementary material for: The telomere bouquet facilitates meiotic prophase progression and exit in fission yeast
Source: Cell Discov. 2017 Nov 7;3:17041–. doi: 10.1038/celldisc.2017.41 (PMC5674143; doi:10.1038/celldisc.2017.41)
Supplement: Supplementary Information [file celldisc201741-s1.pdf]

Supplementary Information

The telomere bouquet facilitates meiotic prophase progression and exit in fission yeast

Vera Moiseeva, Hanna Amelina, Laura C. Collopy, Christine A. Armstrong, Siân R. Pearson, Kazunori Tomita*

INVENTORY

Supplementary Figures

Figure S1 – Mei4 expression and meiotic prophase

Figure S2 – Individual channel images for Figure 1

Figure S3 – Timing of bouquet termination correlates with SPB separation in both wild type and mutants for meiotic recombination

Figure S4 – SPB defects in DNA damage response and bouquet mutants

Figure S5 – *meu13*Δ extends the post-horsetail stage

Figure S6 – SPB settling and Hrs1 foci during the post-horsetail stage

Figure S7 – Mad2 is not involved in the DNA damage checkpoint in the meiotic prophase

Figure S8 – DNA damage Rad52 foci during meiosis

Figure S9 – Remaining numerous RPA foci in *rdh54*Δ originate from meiotic recombination

Figure S10 – Extension of the post-horsetail stage and RPA foci in the DNA damage checkpoint mutants

Figure S11 – Control experiments for the AID system

Figure S12 – Premature termination of the bouquet during meiotic recombination stage

Supplementary Tables

Table S1 – Strains used in this study

Table S2 – Time course data (separate xlsx file)

Supplementary Movies

Movie S1 is the original movie of cell images in Fig. 7a, showing loss of Bqt1-AID at the onset of meiosis I in the absence of auxin.

Movie S2 is the original movie of cell images in Fig. 7b, showing loss of Bqt1-AID during meiotic interphase in the presence of auxin.

Movie S3 is the original movie of cell images in Fig. 8a, showing normal bipolar spindle formation in the absence of auxin.

Movie S4 is the original movie of cell images in Fig. 8b, showing monopolar spindle formation in the presence of auxin.

Movie S5 is the original movie of cell images in Fig. 8c, showing dysfunctional bipolar spindle formation in the presence of auxin.

Supplementary Figures:

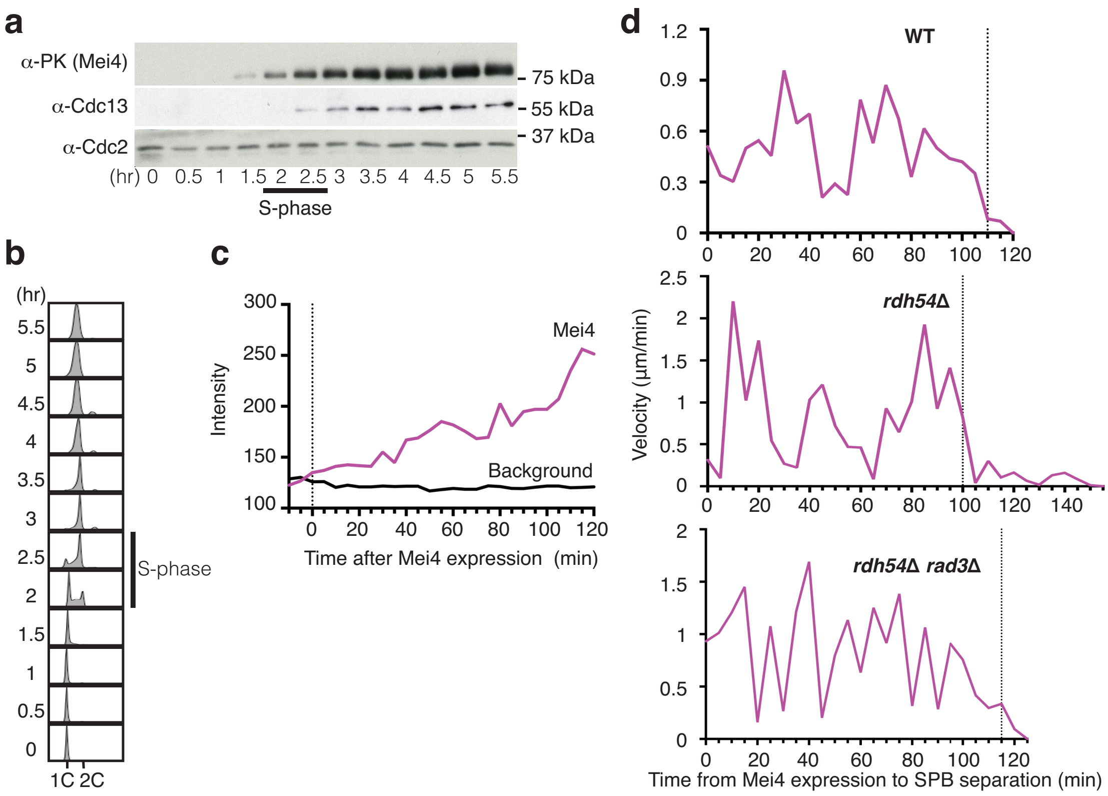

Figure S1 – Mei4 expression and meiotic prophase

(a, b) The *pat1-114* diploid cells were induced to undergo meiosis. Mei4 expression began in pre-meiotic S-phase, as determined by expression timing of cyclin B and DNA content analysis. **(a)** Each fraction was subjected to Western blotting to detect Mei4-PK, Cdc13 (cyclin B) and Cdc2 (CDK1). Cdc13 and Cdc2 were used as a meiotic prophase marker and loading control, respectively. **(b)** Timing of S-phase was determined by DNA content analysis. **(c)** Graph showing signal intensity of mCherry in the nucleus (Mei4, pink line) and cytoplasm (background, blue line) throughout meiotic prophase in a wild type cell shown in Figure 1B. **(d)** Graph showing motion of the SPB throughout meiotic prophase. Analysis of cells shown in Figure 1 is presented. The distance of SPB movement every 5 minutes is measured and represented as velocity (µm/min). The time point of SPB settling is marked as a dotted line (t1).

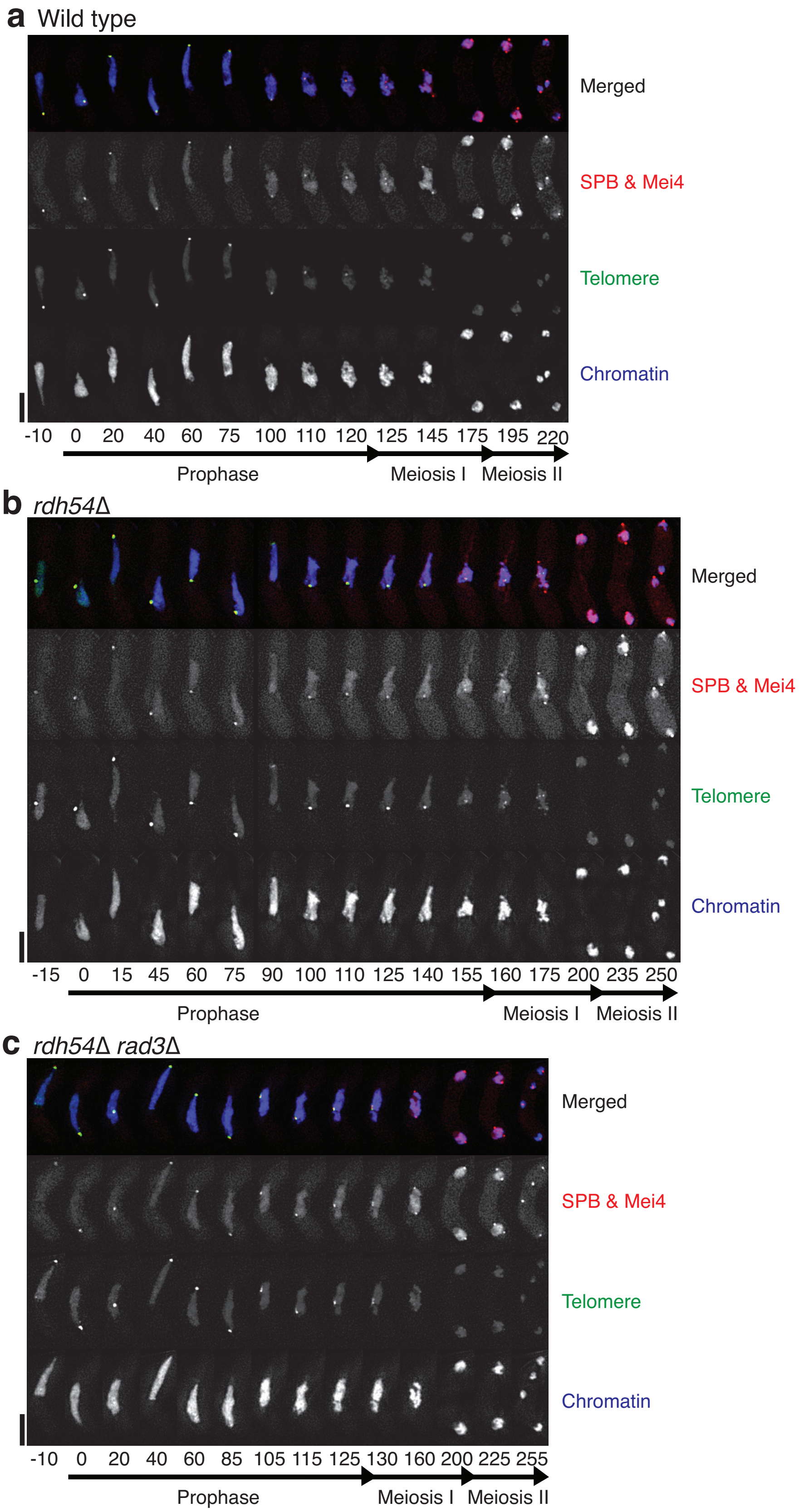

Figure S2 – Individual channel images for Figure 1

(**a-c**) Channels of images from Figure 1b-d are shown, respectively. Numbers below the slides represent minutes after Mei4 staining became visible in the nuclei (t0). Scale bar equals 5 μm.

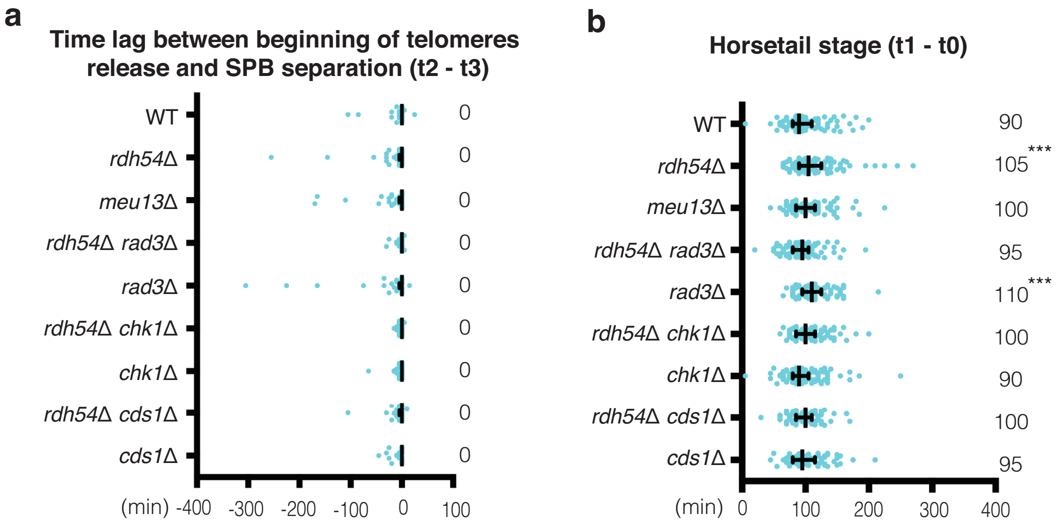

Figure S3 – Timing of bouquet termination correlates with SPB division in both wild type and mutants for meiotic recombination

(a) Distribution graph showing time lag (t2-t3) between telomere dissociation from the SPB (t2) and SPB separation (t3) calculated from dot plots in Figure 1e and 2c. Median durations are indicated on the right. The outside bars represent interquartile range. See Figure 2 for statistics details. The Mann-Whitney nonparametric test suggests that there is no statistically significant difference between the strains (p>0.05). **(b)** Duration of the horsetail stage from Mei4 expression to SPB settling [t1-t0]. Median durations are indicated on the right. The outside bars represent interquartile range. Significant differences over wild type are indicated as asterisks (the Mann-Whitney nonparametric test: *** at $p<0.001$).

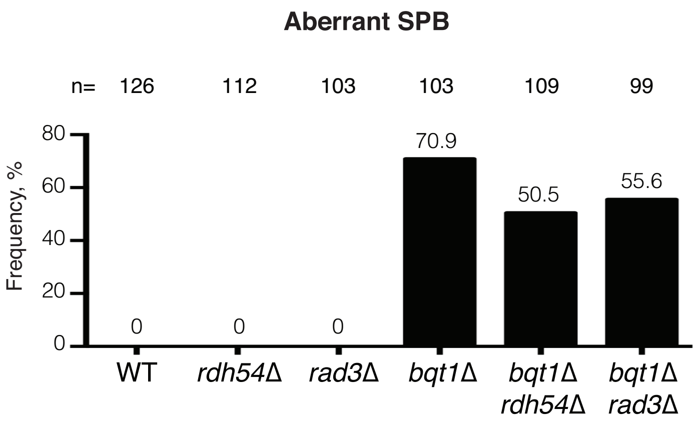

Figure S4 – SPB defects in DNA damage response and bouquet mutants

Graph showing frequency of cells displaying SPB defects. Example image of aberrant SPB in *bqt1Δ* cells is shown in Figure S11.

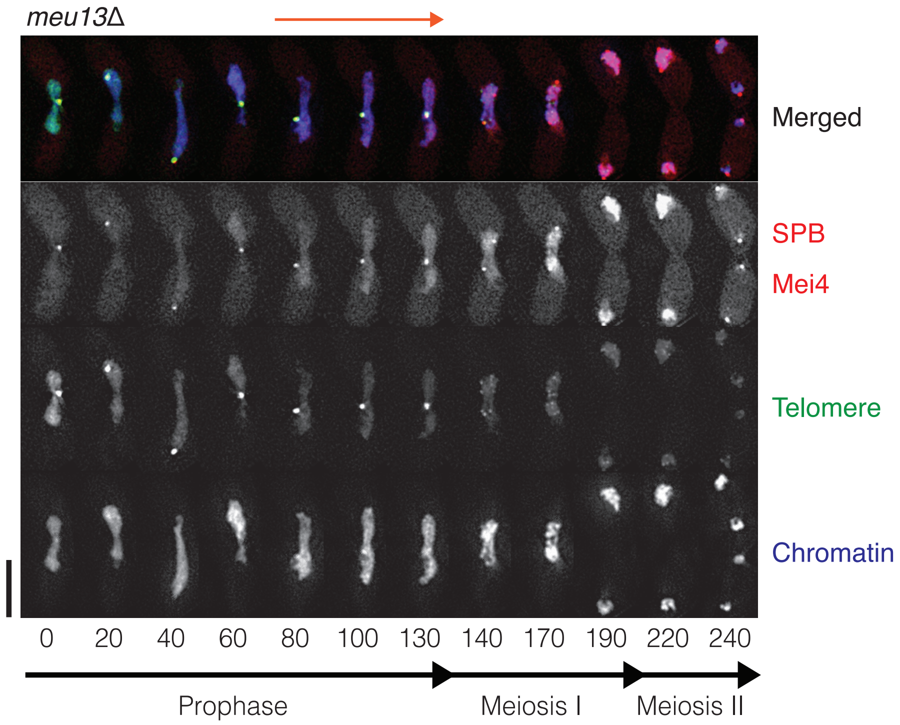

Figure S5 – *meu13*Δ extends the post-horsetail stage

A series of frames from a film of *meu13*Δ cells undergoing meiosis. The SPB, Mei4, telomeres and chromosomes were visualised *via* endogenously tagged Sid4-mCherry, Mei4-mCherry (2nd panel), Taz1-YFP (3rd panel) and Hht1-Cerulean (bottom panel), respectively. Cell images were captured every 5 minutes, and selected time frames are shown. Numbers below the slides represent minutes after Mei4 staining became visible in the nuclei (t0). The post-horsetail stage is highlighted with an orange arrow line above the frames. Scale bar equals 5 µm.

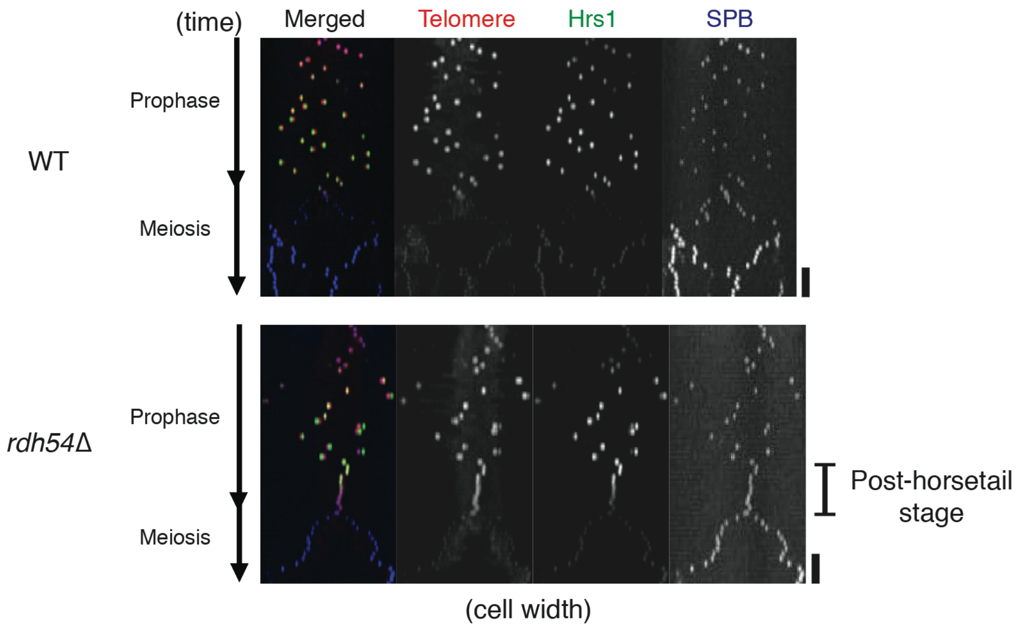

Figure S6 – SPB settling and Hrs1 foci during the post-horsetail stage

Kymograph analysis shows the SPB settling period (the post-horsetail stage). Wild type (top panel) and *rdh54*Δ (bottom panel) cells carrying Taz1-mCherry, Hrs1-YFP and Sid4-Cerulean were filmed every 5 minutes throughout meiosis. Hrs1 is a meiotic prophase specific SPB component, required for SPB oscillation [42]. The kymograph was generated using SoftWoRx 5.5 (Applied Precision). A cell image is aligned to the horizontal axis (x-axis), and is deconvolved and projected using maximum intensity to generate a 2-dimentinal image. The time frames are converted to z-axis. The generated 3-dimentional image (x, y, t) is rotated to (y t, x), and x-axis image is projected using maximum intensity. Resulting images are presented as the position of the foci in x-axis and time point in y-axis. One pixel line of y-axis represents one frame (5 minutes interval) and the scale bar equals 30 minutes. **(Top panel)** Most wild type cells undergo meiosis I once the SPB settles in the middle of the cell. **(Bottom panel)** Conversely, in the majority of *rdh54*Δ cells this SPB settling period is extended, which is highlighted as 'the post-horsetail stage'. Hrs1 signal is gradually degraded during the post-horsetail stage.

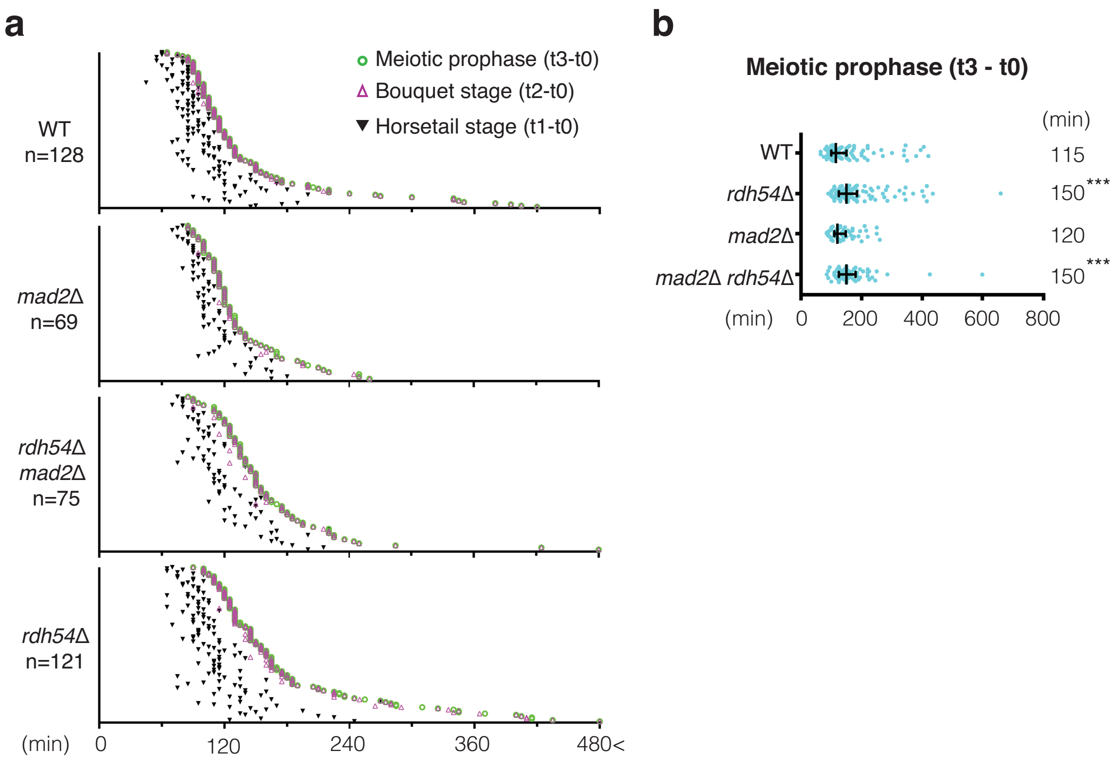

Figure S7 – Mad2 is not involved in the DNA damage checkpoint in the meiotic prophase

(a) Individual dot plots of time distribution of meiotic prophase events in *mad2*Δ and *rdh54*Δ *mad2*Δ cells. Data for WT and *rdh54* Δ are taken from Figure 1e. See the graph in Figure 1e for details. Proportion of the time distributions in *rdh54*Δ *mad2*Δ double mutant cells is reminiscent of that in *rdh54*Δ cells. For values in each sample, see Table S2. **(b)** Graph showing the duration of meiotic prophase (t3-t0), which represents the bouquet stage as well. Data for WT and *rdh54* Δ are taken from Figure 2c. Distribution graphs were calculated from dot plots. Median durations are indicated on the right. The outside bars represent interquartile range. Asterisks indicate statistically significant differences over wild type (the Mann-Whitney nonparametric test: *** at p<0.001).

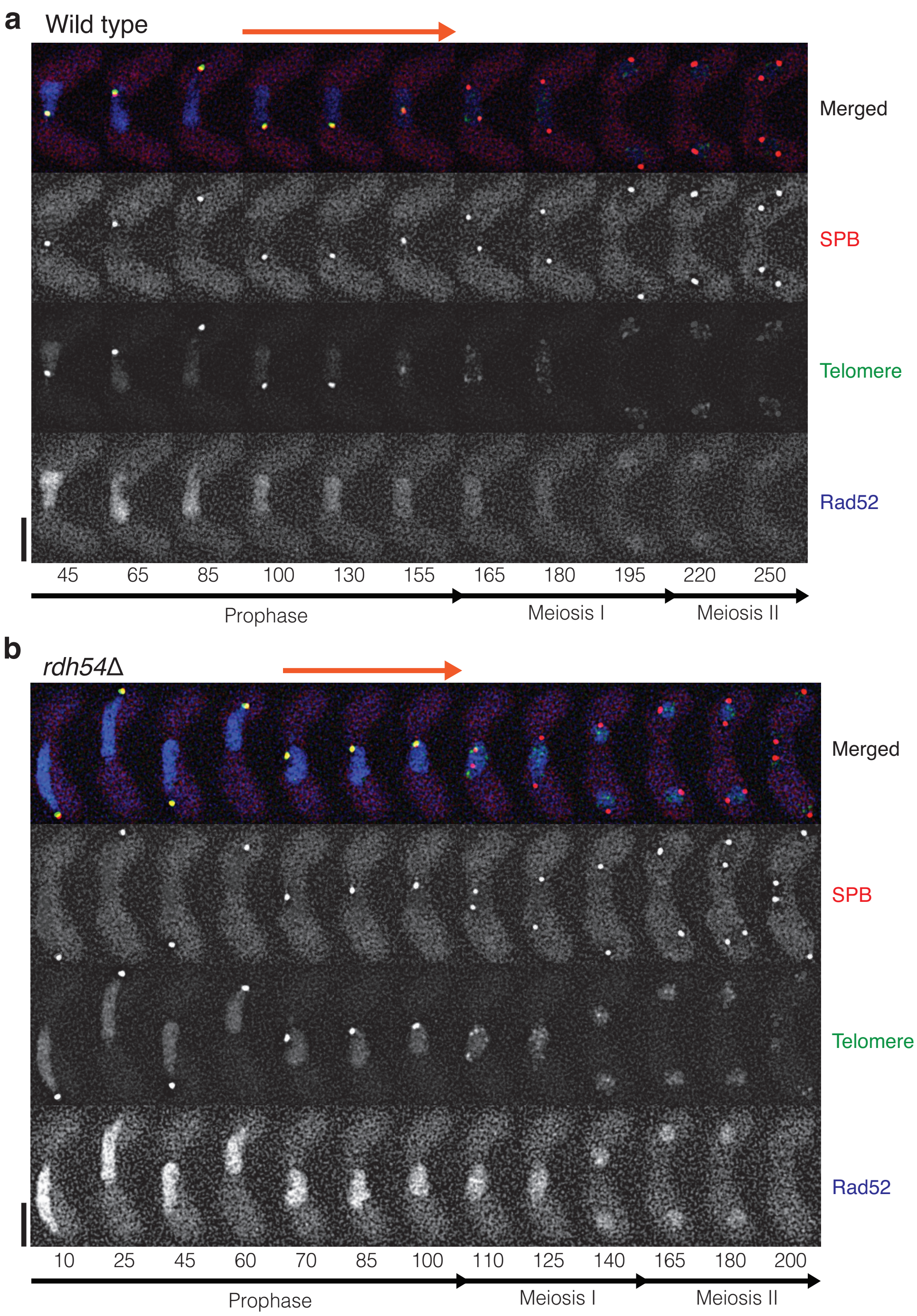

Figure S8 – DNA damage Rad52 foci during meiosis

Series of frames from films of wild type (a) and *rdh54Δ* (b) cells undergoing meiosis. The SPB, telomeres and a DNA damage marker were visualized *via* endogenously tagged Sid4-mCherry (2nd panel), Taz1-YFP (3rd panel) and Rad52-Cerulean (bottom panel), respectively. Cell images were captured every 5 minutes, and selected time frames are shown. Numbers below the slides represent minutes from the beginning of the filming. The post-horsetail stage is highlighted with orange arrow line above the frames. Scale bar equals 5 µm. **(a)** Example images of a wild type cell experiencing a long post-horsetail stage (65 minutes). Rad52 foci are observed during the oscillation phase. Signal intensity of Rad52 is slightly decreased after SPB settling (the 100 minute time point). Forty cells were examined and Rad52 foci were not

observed after a later stage of meiotic prophase. **(b)** In contrast *rad54*Δ cells maintain similar intensity of Rad52 and form distinct foci throughout meiotic prophase. The intensity is decreased at the onset of meiosis I (after the 110 minute time point). Rad52 foci were observed until meiosis II is completed (the 200 minute time point). Nineteen cells were observed and all maintained Rad52 foci during meiotic prophase.

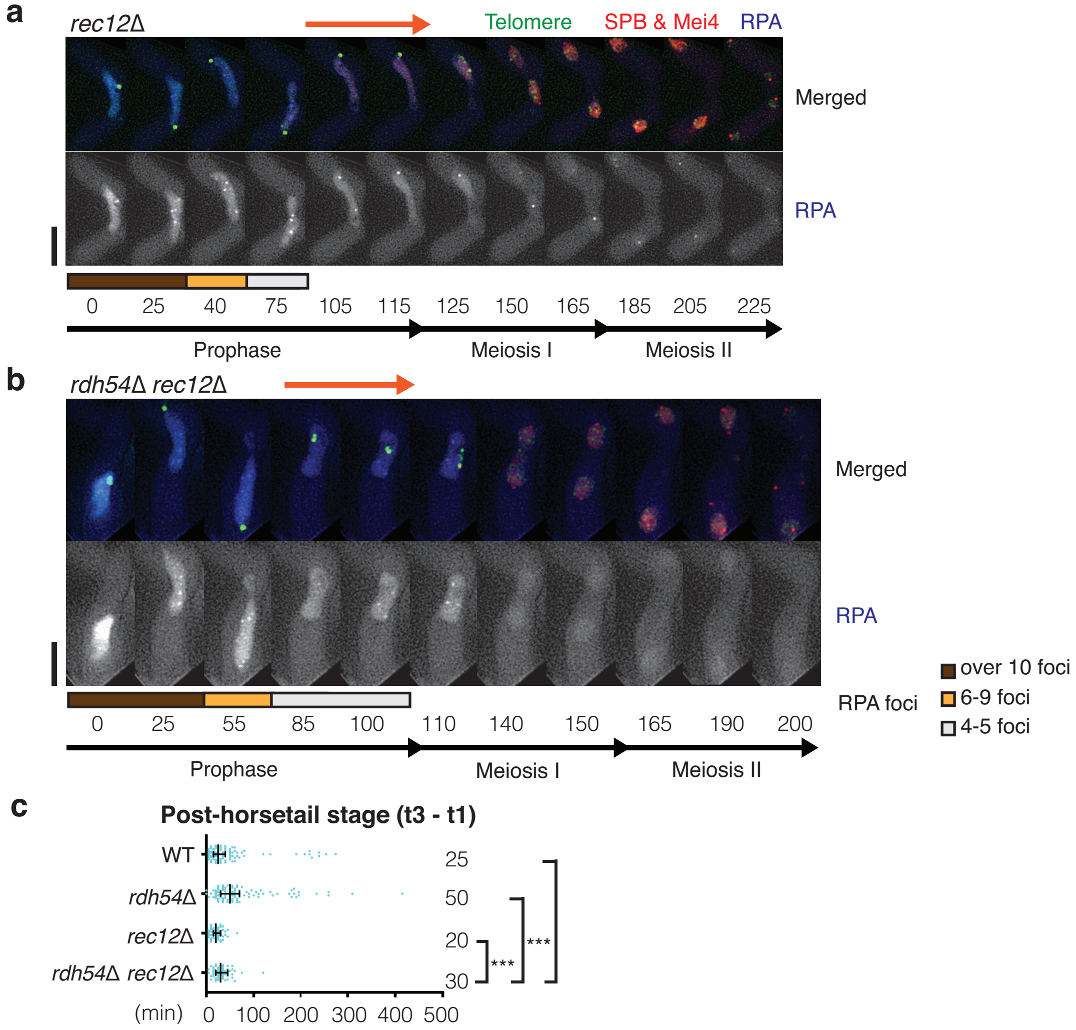

Figure S9 – Remaining numerous RPA foci in *rdh54Δ* originate from meiotic recombination

(a, b) Series of frames from films of cells undergoing meiosis. The SPB, Mei4, telomeres and the RPA component Ssb2 were visualised *via* endogenously tagged Sid4-mCherry, Mei4-mCherry, Taz1-YFP and Ssb2-Cerulean. RPA foci represent sites of chromosome replication and DNA damage (separate channel is shown below merged image). Cell images were captured every 5 minutes, and selected time frames are shown. Numbers below the slides represent minutes after Mei4 staining became visible in the nuclei. The post-horsetail stage is highlighted with orange arrow lines. The coloured bar below the RPA row indicates the number of RPA foci: over 10 foci (brown), 6-9 foci (orange) and 4-5 foci (light grey). Scale bars equal 5 μm. (**a**) An example image of a *rec12Δ* cell is shown. The number of distinct RPA foci decrease in early meiotic prophase but a few foci remain until the post-horsetail stage. In this cell, two RPA foci were detected throughout meiosis. **(b)** Numerous RPA foci shown in *rdh54Δ* single mutant are dependent on the presence of Rec12 that initiates meiotic recombination. The phenotype of *rdh54Δ rec12Δ* double mutant is similar to that of *rec12Δ* single mutant. (**c**) Extension of the post-horsetail stage in *rdh54Δ* is reduced in the absence of Rec12. Distribution graph showing durations of the post-horsetail stage [t3-t1]. Median durations are indicated on the right. Statistically significant differences between the mutants and wild type are indicated as asterisks (two-tailed *t*-test: ** at p<0.01 and *** at p<0.001). Data for WT and *rdh54Δ* are taken from Figure 2b.

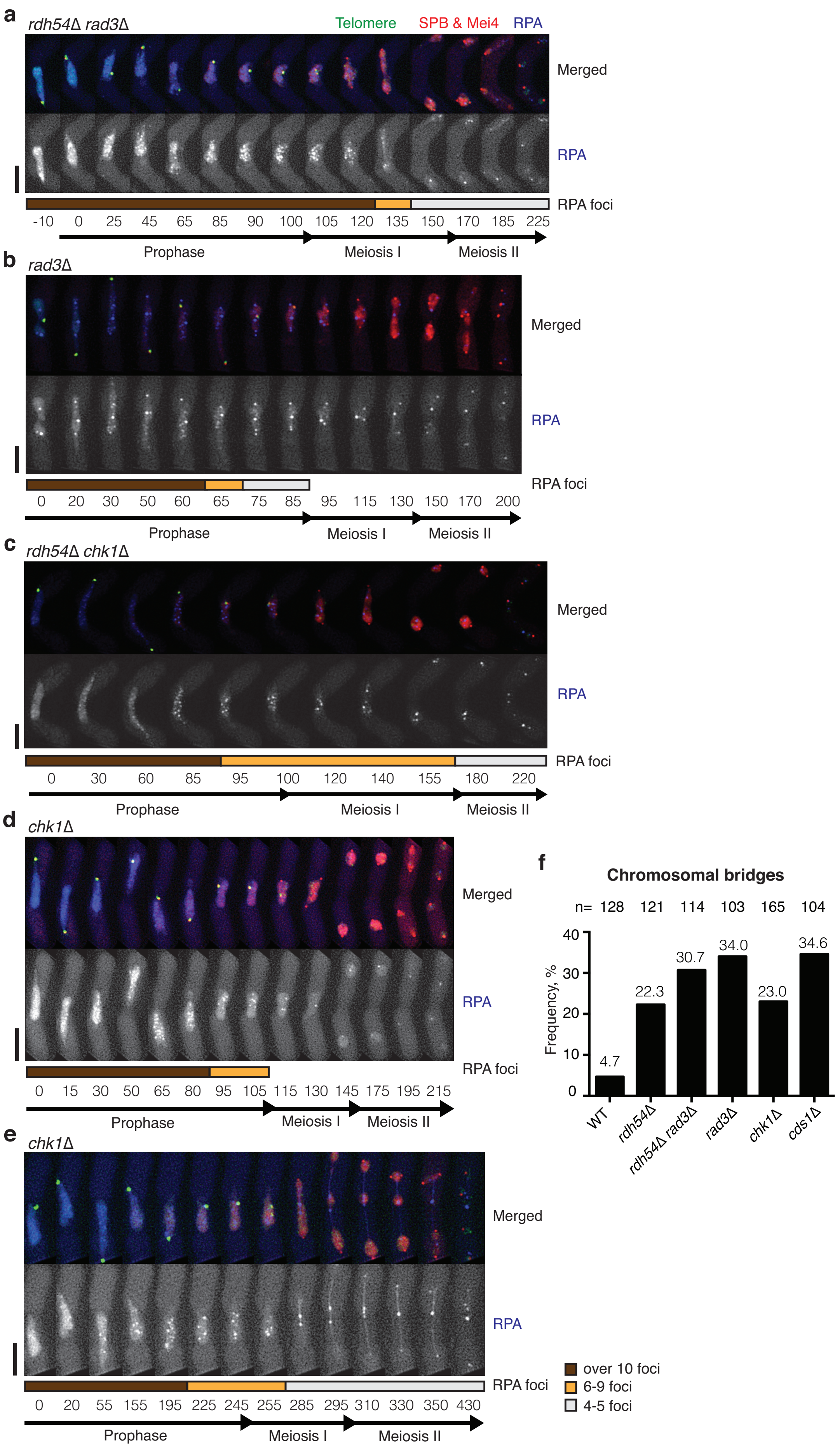

a
rdh54Δ rad3Δ
Telomere
SPB & Mei4
RPA
Merged
RPA
RPA foci
-10 0 25 45 65 85 90 100 105 120 135 150 170 185 225
Prophase
Meiosis I
Meiosis II
b
rad3Δ
Merged
RPA
RPA foci
0 20 30 50 60 65 75 85 95 115 130 150 170 200
Prophase
Meiosis I
Meiosis II
c
rdh54Δ chk1Δ
Merged
RPA
RPA foci
0 30 60 85 95 100 120 140 155 180 220
Prophase
Meiosis I
Meiosis II
d
chk1Δ
Merged
RPA
RPA foci
0 15 30 50 65 80 95 105 115 130 145 175 195 215
Prophase
Meiosis I
Meiosis II
e
chk1Δ
Merged
RPA
RPA foci
0 20 55 155 195 225 245 255 285 295 310 330 350 430
Prophase
Meiosis I
Meiosis II
f
Chromosomal bridges
n= 128 121 114 103 165 104
Frequency, %
0
10
20
30
40
4.7
22.3
30.7
34.0
23.0
34.6
WT
rdh54Δ
rdh54Δ rad3Δ
rad3Δ
chk1Δ
cds1Δ
over 10 foci
6-9 foci
4-5 foci

Figure S10 - Extension of the post-horsetail stage and RPA foci in the DNA damage checkpoint mutants

Series of frames from films of *rdh54*Δ *rad3*Δ (a), *rad3*Δ (b), *rdh54*Δ *chk1*Δ (c) and *chk1*Δ (d) cells undergoing meiosis. The SPB, Mei4, telomeres and RPA were visualised *via* endogenously tagged Sid4-mCherry, Mei4-mCherry, Taz1-YFP and Ssb2-Cerulean, respectively. Cell images were captured every 5 minutes, and selected time frames are shown. Numbers below the slides represent minutes after Mei4 staining became visible in the nuclei (t0). The coloured bar below the RPA row indicates the number of RPA foci: over 10 foci (brown), around 6-9 foci (orange) and 3-5 distinct foci (white). **(a)** *rad3*$^{+}$ deletion diminished the post-horsetail stage of *rdh54*Δ. In this example, the number of RPA foci decreased but were retained through meiotic progression. Fifteen cells were examined and all cells retained significant numbers of RPA foci throughout meiosis, like *rdh54*Δ single mutant cells. However, 3 out of 15 cells exhibited chromosome segregation defects and meiotic catastrophe by retaining over 10 RPA foci during anaphase (data not shown). **(b)** Whereas the post-horsetail stage is shortened, most *rad3*Δ single mutant cells diminish RPA foci before meiosis I and undergo wild type like meiosis (20 out of 23). Three cells retained RPA foci and exhibited chromosome segregation defects (data not shown). **(c)** *rdh54*Δ *chk1*Δ double mutant cells retain RPA foci like *rdh54*Δ *chk3*Δ (n=27). **(d)** *chk1*Δ cells undergo meiosis like *rad3*Δ (n=35). (**e**) Example of chromosome bridge and chromosome loss in *chk1*Δ mutants. (**f**) Graph showing the frequency of chromosome bridges during meiosis.

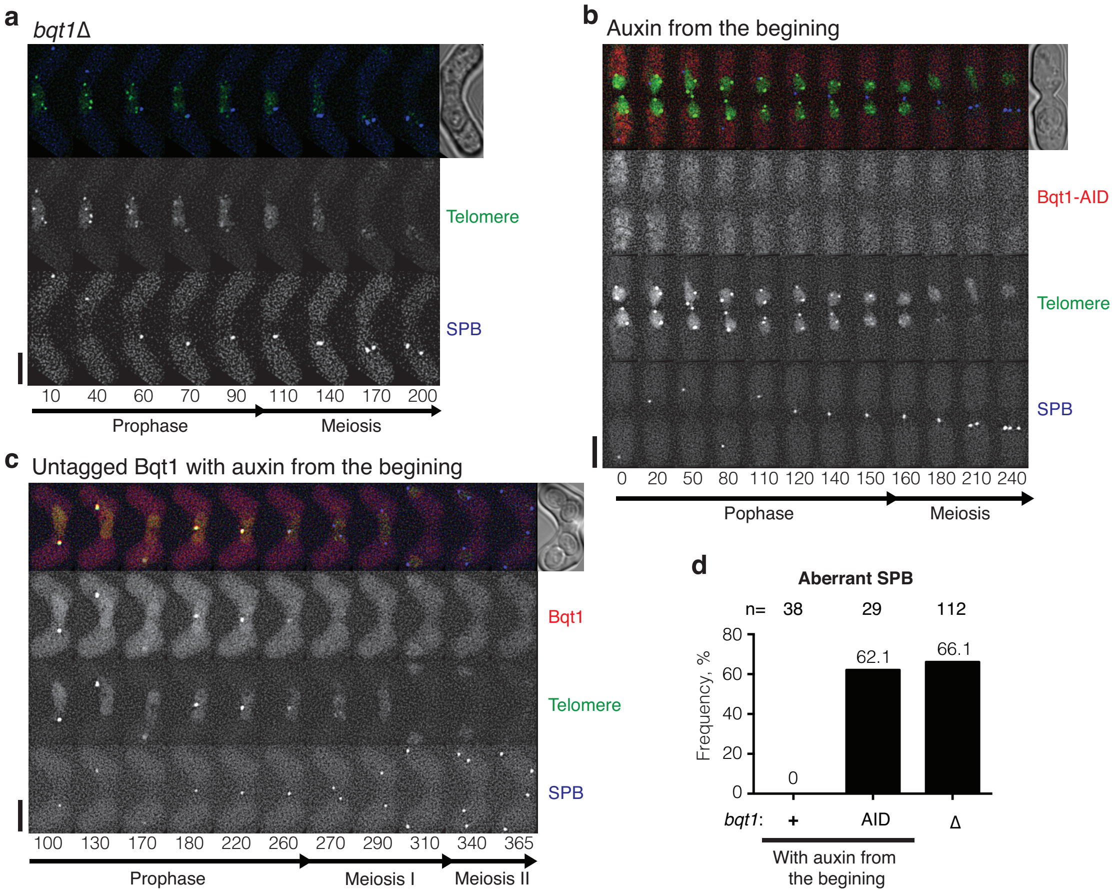

Figure S11 - Control experiments for the AID system

(a-c) Series of frames from films of live fission yeast carrying Rap1-YFP (telomeres) and Sid4-Cerulean (SPB) undergoing meiosis. Cell images were captured every 5 minutes, and selected time frames are shown. Numbers below the slides represent minutes from the beginning of the filming. Spore formation was photographed approximately 12 hours after filming. Scale bars equal 5 μm. **(a)** An example of *bqt1*Δ cell exhibiting de-clustered telomeres and the SPB defects. **(b)** An example of cells carrying SCF^{TIR1} and Bqt1-AID-mCherry in the presence of auxin added at the moment of induction of meiosis. The phenotype represents *bqt1*$^{+}$ deletion. **(c)** An example of cells carrying SCF^{TIR1} and Bqt1-mCherry (no AID tag) in the presence of auxin. **(d)** Graph showing the frequency of SPB defects caused by auxin addition in the indicated *bqt1* genotype strains (+: no AID tagged (c), AID: AID tagged (b), Δ: gene deletion (a)).

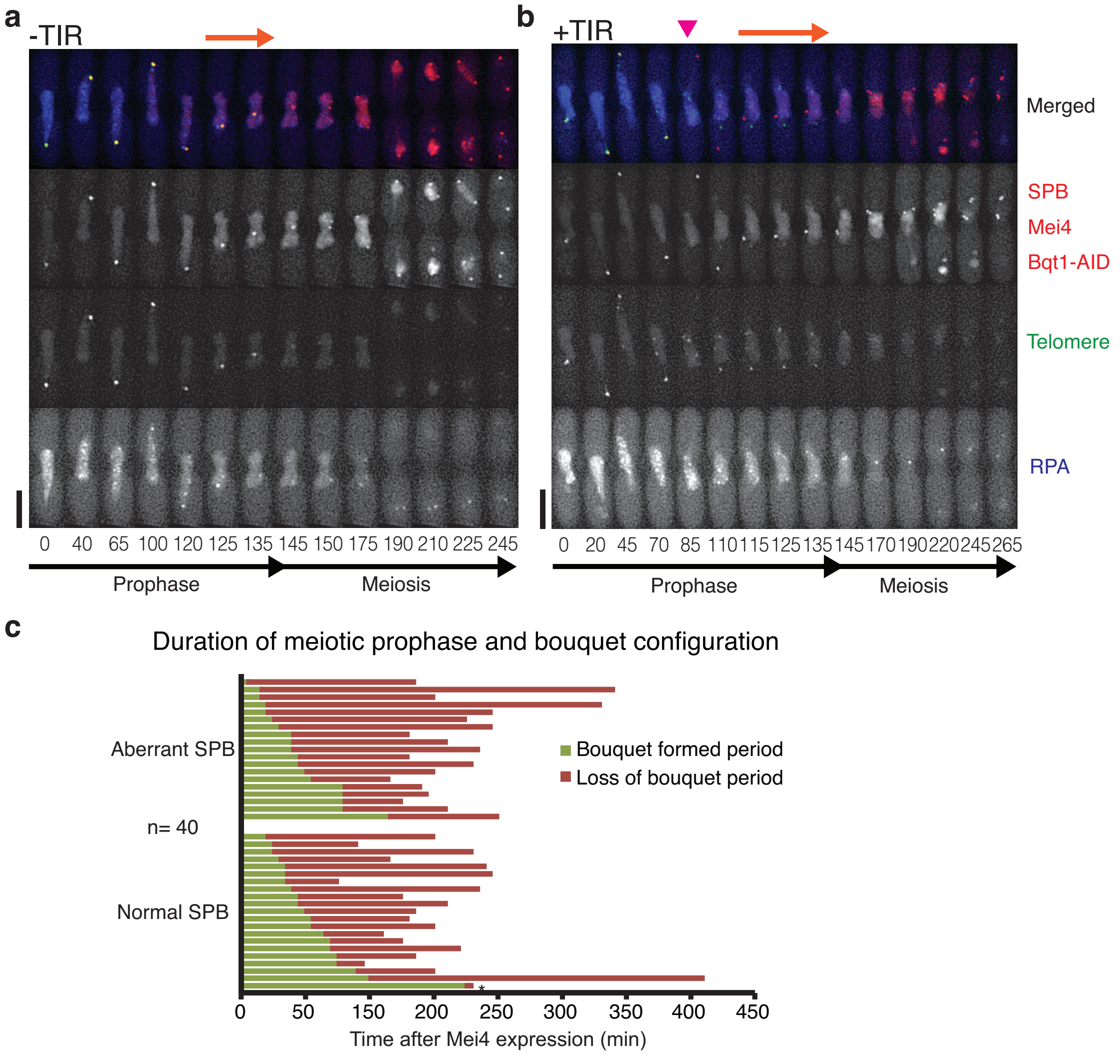

Figure S12 - Premature termination of the bouquet during meiotic recombination stage

(a, b) Series of frames from films of live fission yeast carrying AID-tagged Bqt1 without (a) and with (b) SCF^{TIR1} undergoing meiosis under auxin addition conditions. The SPB, Mei4, Bqt1-AID, telomeres and RPA were visualized *via* endogenously tagged Sid4-mCherry, Mei4-mCherry, Bqt1-AID-mCherry (2nd panel), Taz1-YFP (3rd panel) and Ssb2-Cerulean (bottom panel), respectively. Cell images were captured every 5 minutes, and selected time frames are shown. Numbers below the slides represent minutes after Mei4 staining became visible in the nuclei. Scale bars equal 5 µm. **(a)** Without SCF^{TIR1} expression, telomeres remained clustered at the SPB in the presence of auxin. Telomere dissociation from the SPB followed diminution of RPA foci. A total of 50 cells were observed and all exhibited similar meiotic progress. **(b)** An example of auxin-dependent loss of Bqt1-AID exhibiting SPB defects. The premature termination of the bouquet, represented by dissociation of telomere foci from the SPB, was observed at the 85th minute time point when over 10 RPA foci remained. The number of RPA foci continued to decline until meiotic entry. SPB duplication was observed when RPA foci reduced to three, followed by defective SPB and chromosome segregations. A total of 40 cells were analysed and summarised in the graph (c). **(c)** Duration of the bouquet loss does not correlate with SPB defects. The length of time between Mei4 expression, auxin-dependent loss of termination and the end of meiotic prophase were measured. The duration of bouquet maintenance (green) and loss-of-bouquet periods (red) for

individual cells are shown. Cells were categorized by SPB phenotype (Top: abnormal SPB phenotypes (n=19), Bottom: normal SPB segregations (n=21)). No significant difference was observed in the length of the bouquet configuration between cells with normal and defective SPBs (p=0.3091, two-tailed t-test). 39 cells showed premature termination of the bouquet when more than 5 RPA foci were present. One cell (highlighted by *) showed bouquet termination when RPA foci reduced to 1.

Table S1: Strains used in this study

KTP strain	Genotype
**2390**	*h^{90} ura4-D18 taz1-YFP:kanMX6 hht1-Cerulean:ura4$^+$ Sid4-mCherry:natMX6 mei4-mCherry:natMX6*
**2475**	*h^{90} ura4-D18 rdh54::zeoCV taz1-YFP:kanMX6 hht1-Cerulean:ura4$^+$ Sid4-mCherry:natMX6 mei4-mCherry:natMX6*
**2474**	*h^{90} ura4-D18 meu13::zeoCV taz1-YFP:kanMX6 hht1-Cerulean:ura4$^+$ sid4-mCherry:natMX6 mei4-mCherry:natMX6*
**2902**	*h^{90} ura4-D18 lys1-131 rad3::hygMX6 taz1-YFP:kanMX6 hht1-Cerulean:ura4$^+$ Sid4-mCherry:natMX6 mei4-mCherry:natMX6*
**2708**	*h^{90} ura4-D18 rad3::hygMX6 rdh54::zeoCV taz1-YFP:kanMX6 hht1-Cerulean:ura4$^+$ Sid4-mCherry:natMX6 mei4-mCherry:natMX6*
**3020**	*h^{90} ade6-M216 ura4-D18 chk1::hygMX6 taz1-YFP:kanMX6 hht1-Cerulean:ura4$^+$ Sid4-mCherry:natMX6 mei4-mCherry:natMX6*
**3044**	*h^{90} ade6-M216 ura4-D18 chk1::hygMX6 rdh54::zeoCV taz1-YFP:kanMX6 hht1-Cerulean:ura4$^+$ Sid4-mCherry:natMX6 mei4-mCherry:natMX6*
**3658**	*h^{90} ura4-D18 cds1::zeoCV taz1-YFP:kanMX6 hht1-Cerulean:ura4$^+$ Sid4-mCherry:natMX6 mei4-mCherry:natMX6*
**3657**	*h^{90} ura4-D18 cds1::hygMX6 rdh54::zeoCV taz1-YFP:kanMX6 hht1-Cerulean:ura4$^+$ Sid4-mCherry:natMX6 mei4-mCherry:natMX6*
**3743**	*h^{90} leu1-32 ura4-D18 his3-D1 taz1-YFP:kanMX6 Sid4-mCherry:natMX6 ssb2-Cerulean:kanMX6 mei4-mCherry:ura4$^+$*
**3761**	*h^{90} leu1-32 ura4-D18 his3-D1 rdh54::zeoCV taz1-YFP:kanMX6 Sid4-mCherry:natMX6 ssb2-Cerulean:kanMX6 mei4-mCherry:ura4$^+$*
**3760**	*h^{90} leu1-32 ura4-D18 his3-D1 lys1-131 bqt1::hygMX6 taz1-YFP:kanMX6 Sid4-mCherry:natMX6 ssb2-Cerulean:kanMX6 mei4-mCherry:ura4$^+$*
**3763**	*h^{90} ura4-D18 rad3::hygMX6 taz1-YFP:kanMX6 Sid4-mCherry:natMX6 mei4-mCherry:ura4$^+$ ssb2-Cerulean:kanMX6*
**3762**	*h^{90} ura4-D18 rad3::hygMX6 rdh54::zeoCV taz1-YFP:kanMX6 Sid4-mCherry:natMX6 mei4-mCherry:ura4$^+$ ssb2-Cerulean:kanMX6*
**3774**	*h^{90} leu1-32 his3-D1 ura4-D18 chk1::hygMX6 taz1-YFP:kanMX6 Sid4-mCherry:natMX6 ssb2-Cerulean:kanMX6 mei4-mCherry:ura4$^+$*
**3775**	*h^{90} leu1-32 his3-D1 ura4-D18 chk1::hygMX6 rdh54::zeoCV taz1-YFP:kanMX6 Sid4-mCherry:natMX6 ssb2-Cerulean:kanMX6 mei4-mCherry:ura4$^+$*
**3731**	*h^{90} ura4-D18 bqt1::zeoCV taz1-YFP:kanMX6 hht1-Cerulean:ura4$^+$ Sid4-mCherry:natMX6 mei4-mCherry:natMX6*
**3953**	*h^{90} ura4-D18 bqt1::zeoCV rad3::hygMX6 taz1-YFP:kanMX6 hht1-*

	Cerulean:ura4^{+} Sid4-mCherry:natMX6 mei4-mCherry:natMX6
**4045**	h^{90} ura4-D18 rdh54::zeoCV bqt1::hygML taz1-YFP:kanMX6 hht1-Cerulean:ura4^{+} Sid4-mCherry:natMX6 mei4-mCherry:natMX6
**3292**	h^{90} leu1-32 ura4-D18 ade6:P^{adh15}>skp1-AtTIR1-NLS-9xMyc rap1-YFP:kanMX6 Sid4-Cerulean:natMX6 bqt1::P^{nmt81}>bqt1-3xPK-AID-mCherry:hygMX6
**3432**	h^{90} leu1-32 ura4-D18 ade6:P^{adh15}>skp1-AtTIR1-NLS-9xMyc rap1-YFP:kanMX6 Sid4-Cerulean:natMX6 bqt1::P^{nmt81}>bqt1-3xPK-AID-mCherry:hygMX6 rdh54::ura4^{+}
**3808**	h^{90} leu1-32 ura4-D18 taz1-YFP:kanMX6 ssb2-Cerulean:kanMX6 sid4-mCherry:leu1^{+} mei4-mCherry:ura4^{+} bqt1::Pnmt81>bqt1-3xPK-AID-mCherry:hygMX6
**3863**	h^{90} leu1-32 ura4-D18 taz1-YFP:kanMX6 ssb2-Cerulean:kanMX6 sid4-mCherry:leu1^{+} mei4-mCherry:ura4^{+} ade6^{+}:Padh15>skp1-AtTIR1-2xNLS:natMX6:Padh15>skp1-OsTIR1 bqt1::Pnmt81>bqt1-3xPK-AID-mCherry:hygMX6
**3467**	h^{90} leu1-32 his3-D1 ura4-D18 ade6:P^{adh15}>skp1-AtTIR1-NLS-9xMyc atb2::GFP-atb2:kanMX6 hht1-mRFP:leu1^{+} Sid4-Cerulean:natMX6 bqt1::P^{nmt81}>bqt1-3xPK-AID-mCherry:hygMX6 rdh54::ura4^{+}
**3888**	h^{90} leu1-32 ura4-D18 his3-D1 cdc2-YFP:kanMX6 taz1-mCherry:natMX6 sid4-Cerulean:ura4
**3801**	h^{90} ade6-704 leu1-32 ura4-D18 his3-D1 cdc13-YFP:sup3-5:P^{nmt1}>cdc13 taz1-mCherry:natMX6 sid4-Cerulean:ura4^{+}
**3804**	h^{90} ade6-704 leu1-32 ura4-D18 his3-D1 bqt1::hygMX6 cdc13-YFP:sup3-5:P^{nmt1}>cdc13 taz1-mCherry:natMX6 sid4-Cerulean:ura4^{+}
**4055**	h^{90} ade6-704 leu1-32 ura4-D18 his3-D1 rdh54::zeoCV D1 cdc13-YFP:sup3-5:P^{nmt1}>cdc13 taz1-mCherry:natMX6 sid4-Cerulean:ura4^{+}
**3353**	h^{-}/h^{-} ade6-M210/M216 pat1-114/pat1-114 aur1^{R}:mat-Pc/+ mei4-9xPK:kanMX6/+
**3252**	h^{90} leu1-32 ura4-D18 his3-D1 lys1-131 hrs1-YFP:kanMX6 taz1-mCherry:natMX6 sid4-Cerulean:hygMX6
**3329**	h^{90} leu1-32 ura4-D18 his3-D1 rdh54::zeoCV hrs1-YFP:kanMX6 taz1-mCherry:natMX6 sid4-Cerulean:hygMX6
**3770**	h^{90} ura4-D18 mad2::ura4^{+} rdh54::zeoCV taz1-YFP:kanMX6 hht1-CFP:hygMX6 Sid4-mCherry:natMX6 mei4-mCherry:natMX6
**3230**	h^{90} leu1-32 ura4-D18 his3-D1 taz1-YFP:hygMX6 sid4-mCherry:natMX6 rad52-Cerulean:ura4^{+}
**3259**	h^{90} leu1-32 ura4-D18 his3-D1 rdh54::zeoCV taz1-YFP:hygMX6 sid4-mCherry:natMX6 rad52-Cerulean:ura4^{+}
**3381**	h^{90} ade6-M216 leu1-32 ura4-D18 bqt1::leu1^{+} rap1-YFP:kanMX6 Sid4-Cerulean:ura4^{+}
**3653**	h^{90} leu1-32 ura4-D18 his1-D1 ade6:P^{adh15}>skp1-AtTIR1-NLS-9xMyc rap1-YFP:kanMX6 Sid4-Cerulean:hygMX6 bqt1-mCherry:natMX6
**3769**	h^{90} ura4-D18 taz1-YFP:kanMX6 hht1-CFP:hygMX6 Sid4-mCherry:natMX6 mei4-mCherry:natMX6 mad2::ura4^{+}
**4178**	h^{90} mei4-mCherry:natMX6 taz1-YFP:kanMX6 Sid4-mCherry:natMX6 ssb2-Cerulean:kanMX6 rec12::hygMX6
**4184**	h^{90} mei4-mCherry:natMX6 taz1-YFP:kanMX6 Sid4-mCherry:natMX6 ssb2-Cerulean:kanMX6 rec12::hygMX6
